# Supplementary material for: Cluster randomised controlled trial to determine the effect of peer delivery HIV self-testing to support linkage to HIV prevention among young women in rural KwaZulu-Natal, South Africa: a study protocol
Source: BMJ Open. 2019 Dec 23;9(12):e033435. doi: 10.1136/bmjopen-2019-033435 (PMC7008432; doi:10.1136/bmjopen-2019-033435)
Supplement: Author's manuscript [file bmjopen-2019-033435.draft_revisions.pdf]

BMJ Open

BMJ Open is committed to open peer review. As part of this commitment we make the peer review history of every article we publish publicly available.

When an article is published we post the peer reviewers' comments and the authors' responses online. We also post the versions of the paper that were used during peer review. These are the versions that the peer review comments apply to.

The versions of the paper that follow are the versions that were submitted during the peer review process. They are not the versions of record or the final published versions. They should not be cited or distributed as the published version of this manuscript.

BMJ Open is an open access journal and the full, final, typeset and author-corrected version of record of the manuscript is available on our site with no access controls, subscription charges or pay-per-view fees (http://bmjopen.bmj.com).

If you have any questions on BMJ Open's open peer review process please email info.bmjopen@bmj.com

BMJ Open

A cluster randomised controlled trial to determine the effect of peer delivery HIV Self-Testing to support linkage to HIV prevention among young women in rural KwaZulu-Natal, South Africa: study protocol

Journal:	*BMJ Open*
Manuscript ID	bmjopen-2019-033435
Article Type:	Protocol
Date Submitted by the Author:	06-Aug-2019

Complete List of Authors:	Adeagbo, Oluwafemi; Africa Centre for Health and Population Studies; University of Johannesburg, Sociology<br>Mthiyane, Nondumiso; Africa Centre for Health and Population Studies<br>Herbst, Carina; Africa Centre for Health and Population Studies<br>Seeley, Janet; Africa Centre for Health and Population Studies; London School of Hygiene and Tropical Medicine,<br>Chimbindi, Natsayi; Africa Centre for Health and Population Studies<br>Mee, Paul; London School of Hygiene and Tropical Medicine,<br>Neuman, Melissa; London School of Hygiene and Tropical Medicine,<br>Dreyer, Jaco; Africa Centre for Health and Population Studies<br>Smit, Theresa; Africa Centre for Health and Population Studies<br>Okesola, Nonhlanhla; Africa Centre for Health and Population Studies<br>Johnson, Cheryl; World Health Organization, Department of HIV/AIDS<br>Hatzold, Karin; Population Services International<br>Corbett, Liz; LSHTM, Infectious and Tropical Diseases<br>Cowan, F; Liverpool School of Tropical Medicine, International Public Health; CeSHHAR Zimbabwe,<br>Shahmanesh, Maryam; University College London, Institute for Global Health

Keywords:	HIV/AIDS, Peer delivery model, South Africa, PrEP, ART, HIV Self-testing

SCHOLARONE™
Manuscripts

A cluster randomised controlled trial to determine the effect of peer delivery HIV Self-Testing to support linkage to HIV prevention among young women in rural KwaZulu-Natal, South Africa: study protocol

Adeagbo OA [1,2,4], Mthiyane TN[1], Herbst C[1], Mee P[3], Neuman M[3], Dreyer J[1], Chimbindi N[1,2], Smit T[1]; Okesola N[1], Johnson C[7], HatZold K[8], Seeley J[1,3], Cowan F[5,6], Corbett L[3], Shahmanesh M[1,2]

[1]Africa Health Research Institute, South Africa (formerly known as Africa Centre for Health & Population Studies)

[2]University College London, UK

[3]London School of Hygiene & Tropical Medicine, UK

[4]University of Johannesburg, South Africa

[5] The Centre for Sexual Health and HIV/AIDS Research, Zimbabwe

[6]Liverpool School of Tropical Medicine

[7]World Health Organisation, Switzerland

[8]Population Services International

Corresponding Author: Maryam Shahmanesh, Institute of Global Health, University College London, W61E JB, United Kingdom m.shahmanesh@ucl.ac.uk

ABSTRACT

Introduction:

A cluster randomised controlled trial (cRCT) to determine whether HIV self-testing (HIVST) delivered by peers either directly or through incentivised peer-networks, could increase the uptake of antiretroviral therapy and Pre-Exposure Prophylaxis amongst young women (18-24 years) is being undertaken in an HIV hyperendemic area in KwaZulu-Natal, South Africa.

Methods and analysis:

A 3-arm cRCT started mid-March 2019, in 24 areas in rural KwaZulu-Natal. Twenty-four pairs of peer navigators working with ~12000 young people aged 18-30 over a period of 6 months were randomised to: (1) *incentivised-peer-networks (IPN):* peer-navigators recruited participants "seeds" to distribute up to 5 HIVST packs and HIV prevention information to peers within their social networks. Seeds receive an incentive (20 Rand = $1.5) for each respondent who contacts a peer-navigator for additional HIVST packs to distribute;(2) *peer-navigator-distribution (PND):* peer-navigators distribute HIVST packs and information directly to young people; (3) *standard of care (SOC):* peer-navigators distribute referral slips and information. All arms promote sexual health information and provide barcoded clinic referral slips to

For peer review only - http://bmjopen.bmj.com/site/about/guidelines.xhtml

facilitate linkage to HIV testing, prevention and care services. The primary outcome is the difference in linkage rate between arms, defined as the number of women (18-24 years) per peer-navigators month of outreach work (/pnm) who linked to clinic-based PrEP eligibility screening or started ART, based on HIV-status, within 90 days of receiving the clinic referral slip.

Ethics and dissemination:

This study was approved by the Institutional Review Boards at the World Health Organization, Switzerland (**Protocol ID: STAR CRT, South Africa**), London School of Hygiene and Tropical Medicine, UK (**Reference: 15990-1**), University of KwaZulu-Natal (**BFC311/18**), and the KwaZulu-Natal Department of Health (**Reference: KZ_201901_012**), South Africa. The findings of this trial will be disseminated at local, regional and international meetings and through peer-reviewed publications.

Trial registration number: Clinicaltrials.gov - NCT03751826. Protocol date: 17 January 2019

Keywords: HIV/AIDS; HIV Self-testing; peer delivery model; PrEP; ART; South Africa

XXXXXXXXXXXXXXXXXXXXXXXXXXXXXXXXXXXXXXXXXXXXXXXXXXXXXXXXXXXXXXXXXXXXXXXX

Strengths and limitations of this study

- There is no evidence on the use of HIVST to improve linkage to effective HIV prevention such as Pre-Exposure Prevention.
- There is limited evidence on the strength and limitations of different peer-to-peer approaches to improve uptake of HIV testing and linkage to care and prevention.
- Strengths include the use of a cRCT with rigorous measurement of the outcome linkage to care or prevention by arm, combined with process evaluation and cost-effectiveness studies.
- By embedding this cRCT within a longitudinal demographic surveillance setting, we are able to measure the population reach of the intervention.
- Limitations include a small risk of contamination across clusters and potential for coercive test or intimate partner violence.

XXXXXXXXXXXXXXXXXXXXXXXXXXXXXXXXXXXXXXXXXXXXXXXXXXXXXXXXXXXXXXXXXXXXXXXXX

INTRODUCTION

South Africa has the largest burden of HIV globally with 14% national prevalence rate and an estimated 7.9 million people living with HIV in 2017 [1]. The province of KwaZulu-Natal (KZN) is mostly affected by the epidemic with an 18.1% prevalence rate in 2017[1], while our research setting in uMkhanyakude district has an estimated 30% in the general population [2]. Of the new 88,000 HIV infections recorded amongst young people aged 15-24 years in 2017, 66 000 were among females[1]. Evidence from South Africa and other countries in sub-Saharan Africa shows that most young people living with HIV are undiagnosed and therefore not linked to care [3,4]. Despite an increasing range of effective HIV prevention and treatment

For peer review only - http://bmjopen.bmj.com/site/about/guidelines.xhtml

interventions, including condoms, antiretroviral (ART) based prevention e.g. pre-exposure prophylaxis (PrEP), universal test and treat (UTT) [5,6] and voluntary medical male circumcision (VMMC) there is high HIV incidence rate in adolescent girls and young women (AGYW) with an estimated 5% per annum in aged 15-19 years and 8% per annum in aged 20-24 years respectively in our research setting in Hlabisa sub-district [7]. A recent treatment-as-prevention (TasP) trial conducted in this area failed to show an impact on incidence in part due to the challenge of testing and linking young people and men[8]. Patient level fears (e.g. stigma, labelling and discrimination) and facility level barriers (e.g. distance, waiting times and provider attitudes) continue to be barriers to young people not seeking HIV care services in health facilities [9-11]. There is an urgent need to increase the proportion of those (particularly AGYW) who know their HIV status and take up effective HIV prevention – including ART based care and prevention.

To increase global testing rates and early access to treatment or PrEP, HIV self-testing (HIVST) – which refers to a simple saliva self-test similar to a pregnancy test - has been identified as a potential method given its privacy and convenience [12-14]. Studies have shown high acceptability and uptake of HIVST particularly amongst first time testers and young people [13-17] and A growing number of studies have shown that rapid oral fluid testing was preferred to blood-based testing [18-20]. The OraQuick® In-home HIV test (OraSure Technologies, Inc., Bethlehem, PA, manufactured in Thailand) was recently pre-qualified by the World Health Organization for international procurement[21]. Field based use confirmed the high accuracy of HIVST accuracy, albeit with some variability across different educational levels[14,16,22]. HIVST kit (OraQuick) product is currently available in South Africa and has been endorsed by the National Department of Health (NDoH).

Effective biomedical innovations such as PrEP have the potential to be gamechangers in the HIV epidemic, however, their effectiveness will depend on HIV testing uptake and subsequent linkage to care and prevention [23-25]. The key findings from systematic reviews of the HIV treatment cascade suggest that: (1) community-based delivery models, including adherence clubs, community health workers delivering de-centralised care, and task-shifting to lay caregivers providing support across conditions, improve both ART uptake and sustained retention in low and middle-income settings [26-28]; (2) peer support is effective to deliver health intervention particularly to hard-to-reach groups [29,30]. Moreover, evidence to date suggests that community delivered HIVST supports uptake of testing and linkage to HIV care especially amongst young people [17,31]. However, there is limited data on the role of community based HIVST and/or peer support as a route into HIV prevention.

Here, we describe a cluster randomised controlled trial to address this critical gap in HIVST evidence and linkage for young women aged 18-24 years. The overall aim of this trial is to determine whether HIVST delivered by peers either directly or through incentivised peer-networks, can increase the uptake of antiretroviral therapy (ART) and Pre-Exposure Prophylaxis (PrEP) amongst older adolescent girls and young women at higher risk of HIV (18-24 years) in a high HIV transmission setting in KwaZulu-Natal (KZN), South Africa. . To the best of our knowledge, this is one of the first trials to test the effectiveness of oral-based HIVST to improve uptake of prevention in South Africa.

For peer review only

For peer review only - http://bmjopen.bmj.com/site/about/guidelines.xhtml

Specific Objectives

The specific aims of this trial are to: (1) increase the knowledge of HIV status among young women aged 18-24 years old and their young male partners through the distribution of HIVST through incentivised peer networks or direct distribution by peer navigators compared to peer navigators referring them into HIV testing services; (2) determine an increase in the rate of linkage among young women aged 18-24 years to HIV prevention and treatment services facilitated by distribution of HIVST through incentivised peer networks or direct distribution by peer navigators compared to peer navigators referring into services; (3) determine an overall increase in young men and women aged 18-30 aware of their status and linked to HIV care and prevention; (4) conduct a process evaluation of the acceptability, feasibility, and reach (out of school, recently migrant and living in remote areas) in linking 18-24-year-old women to HIV prevention and treatment services of HIVST distribution through incentivised peer networks, or direct distribution by peer navigators or peer navigators referring into services and (5) measure the cost per 18-24-year-old woman linked to prevention and care through peer-led incentivised HIVST delivery system or direct distribution of HIVST by peer navigators, compared to peer navigator referring into services.

METHODS/DESIGN

Theory of Change

The intervention that is being tested in this cRCT is guided by a theory of change developed through mental models and deductive development[32]. We theorised that the distribution of HIVST kits (including linkage information and referral slips) via peer navigators or peer social networks (respondent driven sampling - RDS) would lead to improved HIV prevention cascade, HIV testing uptake and linkage to HIV treatment or prevention services such as PrEP, amongst young women aged 18-24 years by creating peer-led demand, supporting young people to explore their candidacy for HIV care and prevention in privacy, and using social networks to reach those who need it most[33]. Work done by our group suggested that fear of HIV-related stigma of attending a clinic for HIV testing and discrimination from healthcare providers or community may be addressed by HIVST since individuals can test privately anywhere without fear of being seen or judged[2]. Furthermore, formative work from our group suggested that community based delivery of services through youth friendly and accessible clinics for the study participants (walk-ins and those who present the study referral slips) could provide confirmatory HIV testing, treatment, prevention, contraceptives and other health services[34,35]. We used the SPIRIT reporting guidelines in this article[36].

Trial design

This is a 3-arm cluster randomised controlled trial (two intervention arms and one control arm) launched in mid-March 2019 and currently being carried out by 24 pairs of peer navigators that work with ~12,000 young people aged 18-30 in 24 wards ('Izigodi' in the local language - IsiZulu) in rural uMkhanyakude district of KwaZulu-Natal (KZN), South Africa. This cRCT is comparing two models of peer delivery of HIVST through incentivised respondent driven peer networks and direct distribution by peer navigators compared to standard of care (referral to HIV testing, prevention and care services by peer navigators) in improving the uptake of HIV testing, prevention and care amongst young women (18-24 years)reached by the study arm. Eight (8) pairs of peer navigators were randomised and assigned to each study arm with the intention of reaching 5000 young women aged 18-24 years with HIVST packs (including referral slips) and/or linkage information (including PrEP, contraceptives, ART etc.) during the six-month of community

For peer review only - http://bmjopen.bmj.com/site/about/guidelines.xhtml

outreach. Peer navigators are randomised to 1 of 3 arms: 1) *incentivised-peer-networks:* peer-navigators recruited participants "seeds" to distribute up to 5 HIVST packs and HIV prevention information to peers within social networks. Seeds receive an incentive (20 Rand = $1.5) for each respondent who contacts a peer-navigators for additional HIVST packs to distribute; (2) *peer-navigator-distribution:* peer-navigators distribute HIVST packs and information directly to young people; (3) *standard of care:* peer-navigators distribute referral slips and information. All arms promote sexual health and HIV care and prevention (including PrEP and ART) and provide barcoded clinic referral slips to facilitate linkage to HIV testing, prevention and care services (figure 1).

The unit of randomisation is the pair of peer navigators working in each of the 24 areas included in the study. The areas are not adjoining, and each is bordered by a natural boundary (e.g. roads or streams) or by a sizeable distance. Although contamination is inevitable in this type of cRCT, the spillover effects are contained by measuring the outcome by exposure to the peer-navigator cluster in multiple ways, including barcoded and colour coded referral slips as well as peer-navigator and ward names that determine participant exposure to specific intervention components. Coupled with this, we are conducting a mixed method process evaluation that provides context and add nuance to our understanding of any contamination.

Description of study arms

Intervention arm 1 – incentivised network distribution of HIVST: n=8 pairs of peer navigators are using RDS approach to distribute HIVST packs. Each pair of peer navigators recruits n=10, 18-24 years old female seeds. Each seed fills a brief *service recipient questionnaire* – self-filled on a tablet. Following which they receive a session from the peer-navigator on the HIV prevention services available, the importance of sexual health, the benefits of HIV testing PrEP and ART, and a demonstration of HIVST. Seeds are asked to recruit AGYW aged 18-24 years preferentially but not exclusively and to avoid distribution of -HIVST to those under the age of 18 or over the age of 30. All seeds are asked to complete a brief check of their understanding of the information provided to them, particularly the unreliability of HIVST on ART, the window period, the recommended support to those under 18 using HIVST, and the need for confirmatory testing. As shown in fig. 3 individuals who return the coupons undergo the same procedure as the seeds. They are also given up to 5 uniquely numbered incentivised recruitment coupons and HIVST kits to pass on. When coupons are returned, the original individual who handed out the coupon receives a sum of R20 ($1.5) in airtime per friend or peer who returns the coupon. This sum is a reimbursement for the time that they have spent in explaining and demonstrating the use of an HIVST and is not seen to be an undue incentive to coerce members of their social network to participate. There is no gender restriction of those recruited through the networks, however the primary outcome will be measured in young women aged 18-24 only.

Intervention arm 2: peer navigator direct distribution of HIVST**:** n=8 pairs of peer navigators are distributing HIVST packs directly to young people aged 18-30 years over a period of six months. Each person recruited fills a brief *service recipient questionnaire* – self-filled on a tablet. Following which they receive a session from the peer-navigator on the HIV prevention services available, the importance of sexual health, the benefits of HIV testing, PrEP and ART, and a demonstration of HIV self-screening. All participants are asked to complete a brief check of their understanding of the information provided to

For peer review only - http://bmjopen.bmj.com/site/about/guidelines.xhtml

them, particularly the unreliability of HIVST on ART, the window period, the recommended support to those under 18 using HIVST, and the need for confirmatory testing.

Control - arm 3**:** n=8 pairs of peer navigators are currently distributing linkage information packs and encourage young people aged 18-30 years to test for HIV at clinics, and link to services/care. Each female aged 18-30 approached fills a brief service *recipient questionnaire* – self-filled on a tablet. Following which they receive a session from the peer-navigator on the HIV prevention services available, the importance of sexual health, the benefits of HIV testing PrEP and ART.

Figure 1: Flow diagram of trial enrolment, randomisation and intervention arms

Study oversight

An independent scientific Technical Advisory Group (TAG) was formed by the Unitaid-funded HIV Self-Testing Africa initiative (STAR – a consortium of scientists conducting HIV self-testing related research in different countries) to monitor and supervise progress of data collection, provide independent review of data collected during all cRCTs conducted under the STAR initiative, and assist investigators in disseminating results. TAG comprises members with expertise in HIV epidemiology, statistics, health economics, social science and AGYW. TAG will convey periodic meetings to review data and discuss any issues emanating from this trial.

Setting

This study is taking place in 24 areas in Hlabisa sub-district (estimated 228, 000 population) of uMkhanyakude district in KZN (figure 2). The trial builds on one of the largest population-based HIV incidence cohorts in the world and longstanding research infrastructure in KZN. The study area is mostly rural, and poor compared with other parts of South Africa, with high levels of unemployment and the local language is IsiZulu. The demographic surveillance area provides over 16 years of household history, and over a million person-years of follow-up through annual individual-level surveys, which capture sexual behaviour and partnerships, reproductive histories and contraception use, access to HIV testing and care, access to HIV prevention services (including VMMC), as well as socio-demographic information. The trial will be coordinated from our research office in Somkhele – a rural area in Hlabisa sub-district.

Figure 2: Map of study sites in Hlabisa sub-district in KwaZulu-Natal, South Africa

For peer review only - http://bmjopen.bmj.com/site/about/guidelines.xhtml

Study recruitment and procedures

Peer navigators are a cadre of recently matriculated youth or fresh graduates aged 18-30 years (male and female) recruited from the research community through the local municipal and traditional leaders and they have undergone a rigorous 12-week training in the study procedures and HIVST. The 24 pairs of peer navigators were selected from over 100 youth recruited after several training and assessments. The Peer navigator intervention has been developed in close collaboration with the NDoH and mirrors the South African cadre of community caregivers. We anticipate that the peer navigators working with ~ 12000 young people aged 18-30 will reach ~5000 young women aged 18-24 years in the three-study arms within the six-month of their community outreach work. Before the peer navigators distribute the colour coordinated HIVST packs with unique identifiers in the intervention arms 1 and 2 or information packs in arm 3, the packs are being scanned in and participants are provided with information about the study and fill a brief *service recipient questionnaire* to be completed within Research Electronic Data Capture (REDCap - developed by Vanderbilt University, USA)[37] on a tablet (figure 3). The information is relevant to service delivery only, the date of recruitment, and the ID of peer navigator who recruited them, their age and area of residence. Name, ID (e.g. SA national number), and telephone or WhatsApp contact are optional. Those who are recruited through RDS will also be asked to provide data on their network size, barcode of the RDS coupons and the additional HIVST kits are scanned in. Peer navigators spend ~ 30 minutes with each willing participant to explain the benefits of linking to care and prevention. Those explaining HIVST need 15-20 minutes extra and some young people require more time or more visits. This data is captured in REDCap for the purpose of process evaluation and costing. This data will be used in an aggregate way to understand the process and cost of the service delivery. Individualised data from the survey will only be used in those participants that consent to their clinical data being linked and used for research purposes. If a participant withdraws their consent at any time, their data will be deleted from the research data set.

Participants are being offered study enrollment at the point that they enter any service for the purpose of confirmatory HIV test, and/or eligibility screening for PrEP or HIV treatment. This is being conducted by trained clinical research assistants in mobile and fixed clinics (figure 4). All young women aged 18-24 years coming to one of the 11 primary health care clinics (PHC) or the mobile clinics in the surveillance sites are being directed by our institute data collection clerks to our research nurses. In both settings, the clinical research assistants or the research nurses explain the study and screen the young person for eligibility using a brief eligibility screening questionnaire on REDCap on a tablet. This includes questions to ascertain eligibility as well as arm of the study. If available, the barcode with the unique identifier on the referral slip or the participant's demographic data is used to identify potentially eligible attendees (women aged 18-24 years who have been linked to clinic through the peer navigators). Interested eligible participants are directed to the study research assistant or nurse in a private room who provides information about the study and go through the process of informed consent. Participants provide written informed consent for enrollment into the study and specifically to use the information on their linkage to care as an outcome and to link their baseline questions.

Irrespective of whether they consent to the study, all individuals attending clinics are offered confirmatory HIV testing, with two point of care tests and then blood sent to laboratories for ELISA testing (Genscreen™ ULTRA HIV Ag-Ab qualitative immunoassay [4th Generation] Biorad, Marnes-la-Coquette, France) individuals who test HIV-negative receive counseling around the benefits of PrEP and HIV-positive

For peer review only - http://bmjopen.bmj.com/site/about/guidelines.xhtml

individuals receive counselling around the benefits of immediate starting of ART. If they agree they will undergo clinical screening for PrEP and ART. Screening includes Point of Care (POC) tests for creatinine (StatSensor-I create-test strips, Nova Biomedical UK) to assess renal function and Hepatitis B (Alere Determine™ HBsAg, Alere International Limited, Ballybrit Galway, Ireland; Architect i2000 analyzer, Abbott Diagnostics, Abbott Park, IL, USA), with vaccination offered to those who are negative, and sexual behavior questionnaire to assess eligibility. ART is started by the professional nurses in any of 11 PHCs in the study site. Patients who are eligible for PrEP are started in the three PrEP providing clinics (the mobile vans or the fixed urban adolescent and youth friendly clinic). Persons who are not eligible for PrEP receive counselling and, as indicated, a clinic referral with the screening results. A professional nurse initiates PrEP or ART usually on the same day or within two weeks of the screening visit. The professional nurse provides PrEP counselling that includes (1) *sexual health promotion,* with an emphasis on tackling the multiple health-related behaviors that will affect fertility and sexual pleasure (STIs, mental health, alcohol, diet and exercise); (2) assessment of fertility desire and contraception counselling; (3) choice of contraception and condoms; (4) HIV-negative men are also counselled around the benefits of VMMC and referred accordingly.

The counsellors provide counselling on adherence and develop an individualised adherence plan with the offer of face-to-face or virtual (WhatsApp/ text based) adherence support. If the participant agrees to immediate PrEP initiation, s/he is issued with a month's supply of generic tenofovir disoproxil fumerate and emtricitabine (TDF/FTC). Baseline and follow-up bloods are taken and processed as per SA National Department of Health guidelines. The professional nurse registers the participant at the clinic (or updates the record if the participant is already registered) so that the participant's records are available should the participant seeks care there. Participants receive a phone call seven days after initiating PrEP to complete a standard symptom screen for adverse effects and be referred to clinic for care if necessary. Participants have a clinic appointment scheduled one month, after PrEP initiation, as per national guidelines; appointments for refills and monitoring will be quarterly thereafter through either the mobile clinic, or other community-based refill points. Neutral text message reminders are provided for participants who have access to private messaging and phone calls. Participants are able to reschedule their appointments by text message, WhatsApp or calling the clinical hotline. Contact information is provided for the clinics whom participants can contact at any time.

For peer review only

Inclusion and exclusion criteria

Complete inclusion and exclusion criteria are summarised in tables 1 and 2. There are criteria for different recruitment stages in the trial. Above all, participants must not be less than 18 and not more than 30 years old. They must provide informed consent, not currently on ART and must be living in the study sites.

Table 1: Inclusion and exclusion criteria for receiving the intervention, i.e. the recruitment by Peer Navigators and/or Seeds to receive HIVST packs or clinical referral slips

Inclusion criteria	Exclusion criteria
Participant must not be older than 30 years and younger than 18 years	Participants under 18 years or older than 30 years
Participant must agree to participate	Participant unwilling to participate
Both males and females can be included	None

For peer review only - http://bmjopen.bmj.com/site/about/guidelines.xhtml

Must not be known to be on ART – based on self-report	If on ART

Table 2: Inclusion and exclusion criteria for ascertaining the primary outcome

**Inclusion criteria**	**Exclusion criteria**
Participant must not be older than 24 years and younger than 18 years	Participants under 18 years or older than 24 years
Provide written informed consent	Participants not willing to consent or unable to provide informed consent
Females	Males
Must not be known to be currently on ART	Currently on ART

Table 3: Inclusion and exclusion criteria for ascertaining the secondary outcome

**Inclusion criteria**	**Exclusion criteria**
Participant must not be older than 30 years and younger than 18 years	Participants under 18 years or older than 30 years
Provide written informed consent	Participants not willing to consent or unable to provide informed consent
Must not be known to be currently on ART	Currently on ART

Randomisation

We defined cluster as a pair of peer navigator who live in a same area (Izigodi). All areas in the Southern Population Intervention Platform (PIPSA) of the research locality were selected. Peer navigators were pre-assigned to 24 areas before the randomisation process. Using data from our recent DREAMS (combination HIV prevention) Impact Evaluation study nested within PIPSA, a restricted randomisation was applied to get balanced covariates (location, HIV testing prevalence and uptake of DREAMS combination HIV prevention by adolescent girls and young women) across the 3 arms. We generated a random set of possible 100,000 allocation options. After applying all 3 restrictions, a total of 47, 924 possible allocations remained, and a random number was generated and assigned to each allocation option. The random numbers were ranked from lowest to highest and the allocation option with the first rank was then selected. A randomisation list with intervention arms named alphabetically (A, B & C) was generated.

Following the statistical randomisation, a public randomisation was conducted where peer navigators were divided into three groups (A, B & C). Each group had 16 allocated PNs and 3 floating ones. Each group chose a suitable name and a leader who represented them. Group leads picked a concealed number to determine the order of picking their study arm from a box. The facilitator shook the box so to make sure that each concealed arm in the box had an equal chance of being picked. Lastly, the leaders were asked to open and announce the arms of their respective groups to the bigger group.

Blinding

The statistician and clinical staff did not participate in the public randomisation with peer navigators and they will remain blinded until the results of the study have been finalised.

For peer review only - http://bmjopen.bmj.com/site/about/guidelines.xhtml

Sample size calculations

Based on 2017 data, we estimated ~500 age eligible 18-30 years olds live in each peer navigator team catchment area, of whom we anticipated at least 200, 18-24-year-old females will be handed a study pack (so cluster size at least 200). We estimated this based on 2 peer navigators working approximately 1000 hours over the study period per cluster. We estimated that they would reach 2 young adults per each 4 hour of work and at least one would accept a study pack. We calculated the sample size calculation using the primary outcome, the rate of linkage after 90 days among women ages 18-24 years. Using our existing data on uptake of HIV testing in the DREAMS interventions as well as our data on uptake of testing and linkage to HIV care in the demographic surveillance rounds of, we estimated that 1 woman would link per 6 months of peer navigators outreach work time in the standard of care. With 8 peer educator pairs (or clusters) per arm and a cluster coefficient of variation (k) of 0.25, we will have 80% power to detect a 100% increase in rate from 1 woman to 2 women per 6 months of follow-up, and 90% power to detect a 150% increase from 1 woman to 2.5 women per 6 months of follow-up. We have chosen policy and clinically relevant increases in linkage to care. Assuming additional clustering of the outcome within peer educators and increasing the coefficient of variation (k) to 0.35, we have 80% power to detect a 150% increase in rate from 1 woman to 2.5 women per 6 months of follow-up. All sample size calculations assume two-tailed statistical tests with alpha=0.05.

Outcomes

The long-term goal of the intervention is to increase knowledge of HIV status and improve linkage to HIV care or prevention services such as PrEP amongst young women aged 18-24 years. A number of primary and secondary measures have been defined a priori. An interim analysis of the primary outcome will be conducted at 3 months.

Primary outcome:

The primary outcome is the difference between the rate of linkage of 18-24-year-old women to HIV confirmatory HIV testing, ART (if HIV positive) or PrEP counselling (if HIV negative). The rate is defined as the number of linkages per month of peer navigator outreach activities. The numerator is defined as the number of young women aged 18-24 who attend clinic for confirmatory HIV testing, PrEP counselling or ART, following HIV-ST distribution or peer navigator referral to HIV testing, treatment and prevention services. The denominator for intention-to-treat analysis (ITT) is the entire time (study duration) spent by peer navigators doing their peer outreach work. For the on-treatment analysis, we will use the actual time spent by peer navigators on distributing packs in each arm. The time worked by each peer navigator within a pair will be combined to get the total time per pair of peer navigator. The difference in rate of linkage between the study arms will be calculated - incentivised HIVST delivery through peer network and direct distribution of HIVST will be compared to standard of care.

Secondary outcomes:

The following calculations are planned for the secondary outcomes:

- Comparison of the difference per study arm of the total number of linkages (AGYW aged 18-24) per 100 clinic referral slips distributed.
- Comparison of the difference per study arm of the total number of linkages in men and women aged 18-30 per peer navigator outreach month.

For peer review only - http://bmjopen.bmj.com/site/about/guidelines.xhtml

- The change in proportion of young people aged 18-24 years who are aware of HIVST and who have used HIVST over time.
- Comparison of the difference per study area in the proportion of 18-24-year olds who report knowledge of HIV status and uptake of ART, PrEP and voluntary medical male circumcision (VMMC) in the surveillance area.
- The proportion of hard-to-reach adolescent girls and young women (aged 18-24 years) linked to care in the three arms of study.

Process evaluation

Our aim is to assess the acceptability, feasibility, and fidelity of the peer delivery model in each arm in facilitating linkage to care. We compare the pattern of recruitment per arm and assess the proportion of hard-to-reach AGYW (aged 18-24 years) - defined as out of school, recently migrated and those who live in remote areas linked to care in the three study arms. We also explore potential unintended consequences and ethical issues that arise during peer referral and HIVST and ascertain what works for whom and when to be able to modify the intervention to improve equitable reach and coverage. Specifically, we explore the reach of network recruitment compared to peer outreach work, in terms of reaching more vulnerable groups (out of school, recently migrated, and those who live more remotely). Entrenched in realist evaluation, this process evaluation uses a mixed method approach to investigate implementation, mechanisms of impact and contextual factors, informed by the United Kingdom medical research council (MRC) guide [38] and wider implementation science literature with a focus on fidelity, reach and acceptability [39].

Cost-effectiveness evaluation

We compare the costs in intervention and control arms. Cost per case linked to PrEP eligibility assessment (HIV-) and cost per case started on ART (HIV+). To establish costs, we are using both a bottom-up ingredient-based costing approach and a top-down costing approach using the study budgets and expenditure reports. Specifically, we calculate and cost the actual time spent by peer-navigators in each arm for each person linked to care and prevention.

Data Collection

Participant Survey and Clinic Linkage:

A short survey is administered to consenting individuals participating in the study. The questionnaire collects data on participant's demographic information and voucher identification. The data is captured on REDCap on a tablet. The survey takes approximately 5 minutes to complete and is administered in both English and isiZulu. The primary outcome of linkage is measured through identifying the consenting eligible young women who link to care through the 11 PHCs and the mobile clinics. We use an algorithm to identify which arm the individual came from, including the barcode on the referral slip they bring, the colour of the referral slips or HIVST pack, their area of residence, and the identity of the peer-navigator that recruited them.

Programmatic Data:

In addition to the survey, we collect the programme data records from the peer navigators daily reporting of their outreach activities. This includes the number of young people they have counselled and the numbers they have referred to services and the brief service recipient data they have collected on those

For peer review only - http://bmjopen.bmj.com/site/about/guidelines.xhtml

who have received referral slips or HIVST packs. We use the programme data in an aggregate way (disaggregated only by gender) to understand the reach and coverage of the programme and compare that with those who link to care. We also use data on changes in self-reported HIVST and linkage services collected through the population intervention surveillance platform.

Participant in-depth interviews (IDIs):

IDIs are being conducted with the peer navigators (n= 30), clinical team (n= 6) stationed in clinics in the participating communities and a purposive sample of young women aged 18-24 years (including young men n= 45; 30 females, 15 males) in participating communities and clinics. The interviews are conducted by trained social scientists fluent in English and isiZulu and take approximately 60 minutes in length depending on the participant's responses, and this enables the researchers to understand, contextualize and explore some of the issues emanating from the trial. The small number of IDI participants in qualitative study is allowed since deeper meanings of concepts and thematic areas are explored. To limit disturbances and ensure privacy, the IDIs are conducted in a private space suitable for the participant, and audio recorded with interviewees' consents. Prior to the interview, participants are encouraged to use pseudo names instead of their real names.

Adverse events reporting

HIV testing, including HIVST, is well established and known to have a high level of safety. However, harmful reactions can occur. Adverse events (AE) related to HIVST include all undesirable experiences that result directly from use of the HIVST kit itself or as a reaction from others due to the presence of the kit, use of the kit or results produced from the kit. AEs can be from one person to another, or a person to themselves, and can occur before, during or after self-testing. Also, during PrEP resupply and monitoring visits, participants complete a standardised symptom screening questionnaire for adverse effects of PrEP as per South African clinical guidelines. Furthermore, all participants will receive regular creatinine tests to monitor their renal function. Participants who have severe (grade 3/4) adverse effects and serious adverse effects, are referred to the study clinician for medical evaluation. All participants who experience adverse events receive follow-up until the adverse event is resolved.

AEs and Serious Adverse Events (SAE) are captured through the process evaluation and community engagement units and the telephone hotline. In addition, peer navigators and clinic staff log AEs using our incident reporting form for to up to 12 months after the start of the intervention. Reported AEs and SAEs are monitored, categorised based on an established grading system. SAEs are logged, with the Principal Investigator to evaluate the SAE for seriousness and likely relationship to the intervention. Related SAEs are reported to UKZN and LSHTM Ethics Review Boards. All SAEs are reported regularly through six-month progress reports to TAG members, local and international collaborators. Annual reports with full listings of SAEs will be submitted to Ethics Review Boards.

For peer review only - http://bmjopen.bmj.com/site/about/guidelines.xhtml

Statistical Analysis

The analysis of primary outcome follows an intention-to-treat (ITT) and per-protocol approaches. The analysis of secondary outcomes will be undertaken using per-protocol approach only. Since we randomised the pairs of peer navigator (clusters), the rate of linkage will be calculated for each pair of peer navigator using aggregate data for each cluster. All analyses will be performed using STATA version 15 (StataCorp LP, College Station, Texas USA). In the unadjusted analysis, the mean difference in linkage rate between each intervention arm and standard of care will be assessed using t-test and 95% confidence intervals will be calculated. No adjusted analysis is planned. As part of the exploratory analysis, we will perform a (i) subgroup analysis by gender and area and (ii) two intervention arms will be compared to one another (incentivised HIVST delivery through peer network approach will also be compared to direct distribution of HIVST approach). To expand on this, the data from the client survey captured on REDCap dashboard will be exported into STATA, cleaned and analysed. Descriptive analysis will be performed. Identified variables captured via programmatic monitoring tool will also be exported from the surveillance platform and analysed using STATA to compare data and linkage to care. We will use standard methods for the analysis of cluster randomised trials with small numbers of clusters[40], and reporting will conform to CONSORT guidance for cluster randomised trials[41,42]. Cluster-level summary rates will be calculated and used to estimate the unadjusted rate ratio. Rate ratios adjusting for substantial imbalances in population across arms will also be calculated using a two-stage process. Substantial differences will be identified by comparing frequencies or means of variables known to be associated with the primary outcome. These will be assessed by investigators without the use of statistical tests.

Qualitative analysis

Nvivo software will be used for categorisation and coding of emerging themes from the interview transcripts. Identified themes (including participants' quotes) and interview transcripts will be reviewed and compared by the research team for inconsistencies and adequate representation of participants' views. Emerging themes that address the key focus of the study will be examined and analysed following an interpretivist approach [43].

Data management

Quantitative data are collected directly on the study tablet via REDCap database and resides within a single MySQL database server within a secure server cluster. Study-specific electronic laboratory results are transferred directly to a secured server for storage. Qualitative data are stored in the form of Word files or in Excel both of which can be uploaded into Nvivo qualitative data management programme. The use of MS Word will ensure that data can in future be shared for use in different analysis programmes. These files will be kept on a secure access-controlled folder on a secured server. Qualitative audio files will be destroyed once they have been transcribed, translated and quality controlled.

Patient and public involvement

Although we did not involve patients or the public in the design of the study, findings from previous studies conducted within the community were useful during the study design phase. The study was also presented to the community advisory board (CAB) and the district department of health for comments before it was submitted to IRBs for ethics approval. The results of the study will be shared with the peer

For peer review only - http://bmjopen.bmj.com/site/about/guidelines.xhtml

navigators and the research community through community dialogues and the community advisory board.

Fig 3: Peer Navigators community outreach workflow.

Fig 4: Mobile/Fixed clinics service workflow

ETHICS AND DISSEMINATION

Ethical consideration: confidentiality and informed consent

All staff (including peer navigators) have been provided with training on research ethics such as confidentiality, voluntary participation and good clinical practices. Anonymity and confidentiality are ensured at all levels of the research process, and none of our reports, presentations or articles contain study participants identifying information. Pseudonyms are used when reporting the data particularly qualitative data. Each participant is assigned a unique non-identifying participant identification number. Prior to their involvement in the study, participants are provided with adequate information about the study and are allowed to ask questions for clarifications. Voluntary informed consent is collected only if participants have the full understanding of the study procedures. A copy of the signed consent form is given to them. Participants are informed about the importance of the confirmatory diagnostic testing. An anonymous support hotline is provided on the referral slips should they need to discuss their HIV status, counselling and further linkage to HIV care or other health services. All participant irrespective of their consent to participating in the study are eligible for the clinical services provided through the study. The study was approved by and conforms to the ethical guidelines and standards of UKZN, LSHTM and WHO.

Dissemination plan

The results of this study will be disseminated through traditional academic channels (peer reviewed journal publications) as well as on different information dissemination platforms such as conferences, workshops, community meetings and symposia. The results of the study will also be presented at Self-Testing Africa (STAR) consortium meetings and will be included in the WHO guidelines. The results of the study will be shared with the peer navigators and the research community through community dialogues and the AHRI community advisory board. A detailed findings report will be shared with the Department of Health and other stakeholders to inform policy.

For peer review only - http://bmjopen.bmj.com/site/about/guidelines.xhtml

DISCUSSION

Despite the burden of HIV and the availability of free HIV testing and treatment in our local PHCs, HIV status knowledge remains low amongst young people < 30 years[2]. Several complex barriers (e.g. stigma, confidentiality, family rejection, waiting times and lack of youth friendly services etc.) impeding on young people's access to HIV care services were identified in a formative research we completed in 2018[2]. Studies including systematic reviews[13,17] have shown HIVST to be a promising alternative HIV testing option because it is private, flexible and an efficient method. However, there is limited evidence on the use of HIVST to improve linkage to prevention such as PrEP. The overarching goal of this trial is to address the gap in HIV testing and linkage to HIV prevention among young women aged 18-24 years. This goal will be achieved through the assessment of two HIVST delivery models (incentivized peer network versus direct distribution by peer navigators) compared to the standard of care of peer navigator only.

A major strength of this study is the development of a theoretically derived intervention that can be implemented through existing cadre of community caregivers and peer to peer networks across SSA[44-47]. If found to be effective in increasing HIV testing uptake and prevention, the intervention is designed to be rolled out. Also, using a rigorous cost effectiveness analysis will allow SA policy makers to evaluate the cost-benefit ratio of using the different models of distribution in different settings. Furthermore, by collecting rigorous data on linkage to prevention both through the trial and from our surveillance infrastructure, we can understand the potential population impact of the different methods of HIVST distribution on knowledge of status and linkage to care and prevention. Ultimately this will provide evidence of the potential of the intervention to attract young people into the HIV care and prevention cascade and inform the evidence base to reduce the mortality and morbidity in youth. Lastly, rigorous process evaluation and collection of data on all adverse events and social harms will provide important data around some of the concerns about HIVST, i.e. the potential for coercive test, depression, anxiety, suicidality or intimate partner violence.

In order to reduce the risk of contamination due to the proximity of the areas and the nature of the intervention, we have piloted several methods including the use of referral slips with unique codes and colour coordinated packs to identify the arm individuals come from when they link to care. Previous data from nested cohorts that we have followed up in our area has shown that young people rarely migrate within our surveillance area given that only 220 of 2 184 young people < 25 years cohorts moved from one cluster to another in 2017/2018. Furthermore, the process evaluation will help us understand the delivery model in each arm as well as unintended consequences and ethical issues that arise during the study and ascertain what works for whom and when to be able to modify the intervention to improve equitable reach and coverage.

In conclusion, the results of this trial are expected to contribute to WHO guidelines and informed policy aimed at implementation and scale-up of HIVST and PrEP in South Africa. Also, this study will address critical gaps in the literature on HIV testing and prevention interventions for young people particularly females aged 18-24 years in Southern Africa.

Acknowledgements:

The authors acknowledge TAG of the STAR initiative and AHRI HIV Prevention Multilevel Group including the research assistants, peer navigators, clinical team, managers and research administrators for their

For peer review only - http://bmjopen.bmj.com/site/about/guidelines.xhtml

commitment to the study. We also extend our appreciation to our research community including the community advisory boards in uMkhanyakude district.

Authors Contributions:

MS and LC conceived the study. OA, MS, CH, JD, JS, LC, FC, NC, NO, PM, MTN MN designed the study. OA and MS wrote the first draft of the manuscript. OA, MS, LC, JS, JD, CH, TS, FC, MN, NC, KH, CJ, PM, MTN, and NO read and critically revised the manuscript. All authors read and approved the final manuscript.

Funding:
This study is part of the Self-Testing Africa (STAR) initiative funded by the Unitaid (grant number: PO#10140-0-600). MS is partly supported by the US National Institute of Health (NIH) R01 (award no: 5R01MH114560-03) that supports a peer led outreach team of navigators to support uptake and retention of adolescents and young adults in existing HIV prevention. Africa Health Research Institute is supported by core funding from the Wellcome Trust [Core grant number (082384/Z/07/Z)]. The funders were not involved in the design of the study.

Competing interests:
None declared.

Ethics approval:

Ethical approval was obtained for the study from Institutional Review Boards (IRB) of the World Health Organisation, Switzerland (**Protocol ID: STAR CRT, South Africa**), London School of Hygiene and Tropical Medicine, UK (**Reference no: 15990 - 1**), University of KwaZulu-Natal (**BFC311/18**), and the KwaZulu-Natal Department of Health (**NHRD Reference no: KZ_201901_012**) , South Africa. The study was also approved by the community advisory board (CAB) representing the research community before it was submitted to IRBs. ***Trial registration number***: Clinicaltrials.gov - NCT03751826. Protocol date: 17 January 2019.

Data sharing statement:
On completion of the trial, de-identified data will be available through the Africa Health Research Institute, South Africa (AHRI) data access repository upon request. Requests for data sharing are reviewed by the data custodian and a Data Sharing committee at AHRI. After approval, all data sharing, even those without identifiers, will be through a secure and password-controlled data sharing site, with access strictly limited to transcripts that are relevant to the analysis planned. Users will be asked to abide by the AHRI Data Access and Sharing Policy.

For peer review only - http://bmjopen.bmj.com/site/about/guidelines.xhtml

REFERENCES

1. HSRC. *South African National HIV Prevalence, Incidence, Behaviour and Communication Survey 2017*

Pretoria, South Africa: Human Science Research Council July, 2018 2018.

2. Adeagbo O, Herbst C, Dlamini N, et al. The 'Missing Middle' in HIV Treatment Cascade: Ensuring Men's and Young People's access to HIV Care in KwaZulu-Natal, South Africa. Association of Social Science and Humanities in HIV , 2018 International AIDS Society Pre-conference 21 July 2018, 2018; Amsterdam.
3. Huerga H, Van Cutsem G, Farhat JB, et al. Progress towards the UNAIDS 90–90-90 goals by age and gender in a rural area of KwaZulu-Natal, South Africa: a household-based community cross-sectional survey. *BMC public health.* 2018;18(1):303.
4. Carrasco MA, Fleming P, Wagman J, Wong V. Toward 90-90-90: identifying those who have never been tested for HIV and differences by sex in Lesotho. *AIDS care.* 2018;30(3):284-288.
5. Organization WH. WHO expands recommendation on oral preexposure prophylaxis of HIV infection (PrEP): Policy brief. *WHO, Geneva.* 2015.
6. (NDoH) NDoHSA. *National Policy on HIV Pre Exposure Prophylaxis (PrEP) and test and treat (T&T).* Pretoria, South Africa2016.
7. M S, N C, N M, et al. Persistently high incidence of HIV in adolsescent girls and young women in rural KwaZulu-Natal, South Africa: Lessons for Determined Resilient, Empowered, AIDS free, Mentored and Safe (DREAMS) multilevel HIV prevention scale-up. Paper presented at: AIDS Impact; 17 November 2017, 2017; Cape Town, South Africa.
8. Iwuji C, Orne-Gliemann J, Larmarange J, et al. Universal test and treat and the HIV epidemic in rural South Africa: a phase 4, open-label, community cluster randomised trial. *The lancet HIV.* 2018;5(3):e116-e125.
9. Plazy M, Farouki KE, Iwuji C, et al. Access to HIV care in the context of universal test and treat: challenges within the ANRS 12249 TasP cluster-randomized trial in rural South Africa. *Journal of the International AIDS Society.* 2016;19(1):20913.
10. Moshabela M, Zuma T, Orne-Gliemann J, et al. "It is better to die": experiences of traditional health practitioners within the HIV treatment as prevention trial communities in rural South Africa (ANRS 12249 TasP trial). *AIDS care.* 2016;28(sup3):24-32.
11. Boyer S, Iwuji C, Gosset A, et al. Factors associated with antiretroviral treatment initiation amongst HIV-positive individuals linked to care within a universal test and treat programme: early findings of the ANRS 12249 TasP trial in rural South Africa. *AIDS care.* 2016;28(sup3):39-51.
12. Spielberg F, Levine RO, Weaver M. Self-testing for HIV: a new option for HIV prevention? *The Lancet infectious diseases.* 2004;4(10):640-646.
13. Krause J, Subklew-Sehume F, Kenyon C, Colebunders R. Acceptability of HIV self-testing: a systematic literature review. *BMC public health.* 2013;13(1):735.
14. Choko AT, Desmond N, Webb EL, et al. The uptake and accuracy of oral kits for HIV self-testing in high HIV prevalence setting: a cross-sectional feasibility study in Blantyre, Malawi. *PLoS medicine.* 2011;8(10):e1001102.
15. Spielberg F, Critchlow C, Vittinghoff E, et al. Home collection for frequent HIV testing: acceptability of oral fluids, dried blood spots and telephone results. *Aids.* 2000;14(12):1819-1828.
16. Belza MJ, Rosales-Statkus ME, Hoyos J, et al. Supervised blood-based self-sample collection and rapid test performance: a valuable alternative to the use of saliva by HIV testing programmes with no medical or nursing staff. *Sex Transm Infect.* 2012:sextrans-2011-050131.

For peer review only - http://bmjopen.bmj.com/site/about/guidelines.xhtml

17. Harichund C, Karim QA, Kunene P, Simelane S, Moshabela M. Exploring factors that influence the integration of HIVST with HCT using a qualitative comparative cross-over design in KwaZulu-Natal, South Africa. *Global public health.* 2019:1-13.
18. Gaydos CA, Hsieh Y-H, Harvey L, et al. Will patients “opt in” to perform their own rapid HIV test in the emergency department? *Annals of emergency medicine.* 2011;58(1):S74-S78.
19. Kalibala S, Tun W, Muraah W, Cherutich P, Oweya E, Oluoch P. Knowing myself first’: feasibility of self-testing among health workers in Kenya. *Nairobi: Population Council.* 2011.
20. Lee VJ, Tan SC, Earnest A, Seong PS, Tan HH, Leo YS. User acceptability and feasibility of self-testing with HIV rapid tests. *JAIDS Journal of Acquired Immune Deficiency Syndromes.* 2007;45(4):449-453.
21. WHO. *Guidelines on HIV self-testing and partner notification.* Switzerland: WHO;2016.
22. Kapaku K, Neuman M, Maluzi K, et al. Is OraQuick HIV-self-testing valid among intended users? Analysis from a clinical performance study in Lusaka, Zambia. *International AIDS Society (IAS 2017) Paris, France.* 2017.
23. Van Damme L, Corneli A, Ahmed K, et al. Preexposure prophylaxis for HIV infection among African women. *New England Journal of Medicine.* 2012;367(5):411-422.
24. Marrazzo JM, Ramjee G, Richardson BA, et al. Tenofovir-based preexposure prophylaxis for HIV infection among African women. *New England Journal of Medicine.* 2015;372(6):509-518.
25. Yun K, Xu J-j, Zhang J, et al. Female and younger subjects have lower adherence in PrEP trials: a meta-analysis with implications for the uptake of PrEP service to prevent HIV. *Sex Transm Infect.* 2017:sextrans-2017-053217.
26. Kim S-H, Gerver SM, Fidler S, Ward H. Adherence to antiretroviral therapy in adolescents living with HIV: systematic review and meta-analysis. *AIDS (London, England).* 2014;28(13):1945.
27. Sharma M, Barnabas RV, Celum C. Community-based strategies to strengthen men’s engagement in the HIV care cascade in sub-Saharan Africa. *PLoS medicine.* 2017;14(4):e1002262.
28. Van Rooyen H, Barnabas RV, Baeten JM, et al. High HIV testing uptake and linkage to care in a novel program of home_based HIV counseling and testing with facilitated referral in KwaZulu-Natal, South Africa. *Journal of acquired immune deficiency syndromes (1999).* 2013;64(1):e1.
29. Kanters S, Park JJ, Chan K, et al. Interventions to improve adherence to antiretroviral therapy: a systematic review and network meta-analysis. *The Lancet HIV.* 2017;4(1):e31-e40.
30. Pettifor A, Nguyen NL, Celum C, Cowan FM, Go V, Hightow-Weidman L. Tailored combination prevention packages and PrEP for young key populations. *Journal of the International AIDS Society.* 2015;18(2S1).
31. Choko AT, MacPherson P, Webb EL, et al. One Year Outcomes Following Community-Based HIV Self-Testing: A Prospective Study in Malawi. Conference on Retroviruses and Opportunistic Infections 2014; United States.
32. Funnell SC, Rogers PJ. *Purposeful program theory: Effective use of theories of change and logic models.* Vol 31: John Wiley & Sons; 2011.
33. Hargreaves JR, Delany-Moretlwe S, Hallett TB, et al. The HIV prevention cascade: integrating theories of epidemiological, behavioural, and social science into programme design and monitoring. *Lancet HIV.* 2016;3(7):e318-322.
34. Adeagbo O, Herbst C, Dlamini N, et al. " If She Tests Negative, It Means I Am Also Negative": Men's Construction of HIV Testing in South Africa. *AIDS RESEARCH AND HUMAN RETROVIRUSES.* 2018;34:85-85.
35. Nkosi B, Seeley J, Ngwenya N, et al. Exploring adolescents and young people’s candidacy for utilising health services in a rural district, South Africa. *BMC Health Services Research.* 2019;19(1):195.

For peer review only - http://bmjopen.bmj.com/site/about/guidelines.xhtml

36. Chan A-W, Tetzlaff JM, Altman DG, et al. SPIRIT 2013 statement: defining standard protocol items for clinical trials. *Annals of internal medicine.* 2013;158(3):200-207.
37. Harris PA, Taylor R, Thielke R, Payne J, Gonzalez N, Conde JG. Research electronic data capture (REDCap)—a metadata-driven methodology and workflow process for providing translational research informatics support. *Journal of biomedical informatics.* 2009;42(2):377-381.
38. Moore GF, Audrey S, Barker M, et al. Process evaluation of complex interventions: Medical Research Council guidance. *bmj.* 2015;350:h1258.
39. Jacobs C, Michelo C, Moshabela M. Implementation of a community-based intervention in the most rural and remote districts of Zambia: a process evaluation of safe motherhood action groups. *Implementation science.* 2018;13(1):74.
40. Hayes RJ, Moulton LH. *Cluster randomised trials.* Chapman and Hall/CRC; 2017.
41. Campbell MK, Piaggio G, Elbourne DR, Altman DG. Consort 2010 statement: extension to cluster randomised trials. *Bmj.* 2012;345:e5661.
42. Campbell MK, Elbourne DR, Altman DG. CONSORT statement: extension to cluster randomised trials. *Bmj.* 2004;328(7441):702-708.
43. Denzin NK, Lincoln YS. *The Sage handbook of qualitative research.* Sage; 2011.
44. Onyango MA, Adu-Sarkodie Y, Agyarko-Poku T, et al. “It's all about making a life”: poverty, HIV, violence, and other vulnerabilities faced by young female sex workers in Kumasi, Ghana. *JAIDS Journal of Acquired Immune Deficiency Syndromes.* 2015;68:S131-S137.
45. Geibel S, King'ola N, Temmerman M, Luchters S. The impact of peer outreach on HIV knowledge and prevention behaviours of male sex workers in Mombasa, Kenya. *Sex Transm Infect.* 2012;88(5):357-362.
46. Oldenburg CE, Biello KB, Colby D, et al. Engagement with peer health educators is associated with willingness to use pre-exposure prophylaxis among male sex workers in Ho Chi Minh City, Vietnam. *AIDS patient care and STDs.* 2014;28(3):109-112.
47. Ford K, Wirawan D, Suastina W, Reed B, Muliawan P. Evaluation of a peer education programme for female sex workers in Bali, Indonesia. *International journal of STD & AIDS.* 2000;11(11):731-733.

For peer review only

For peer review only - http://bmjopen.bmj.com/site/about/guidelines.xhtml

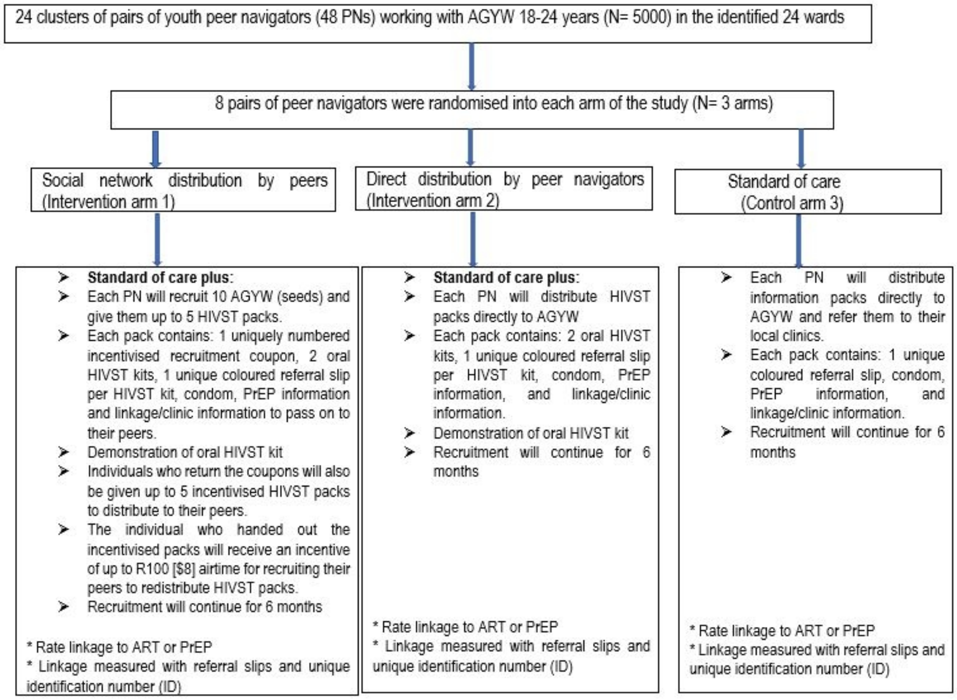

Figure1_300

66x48mm (300 x 300 DPI)

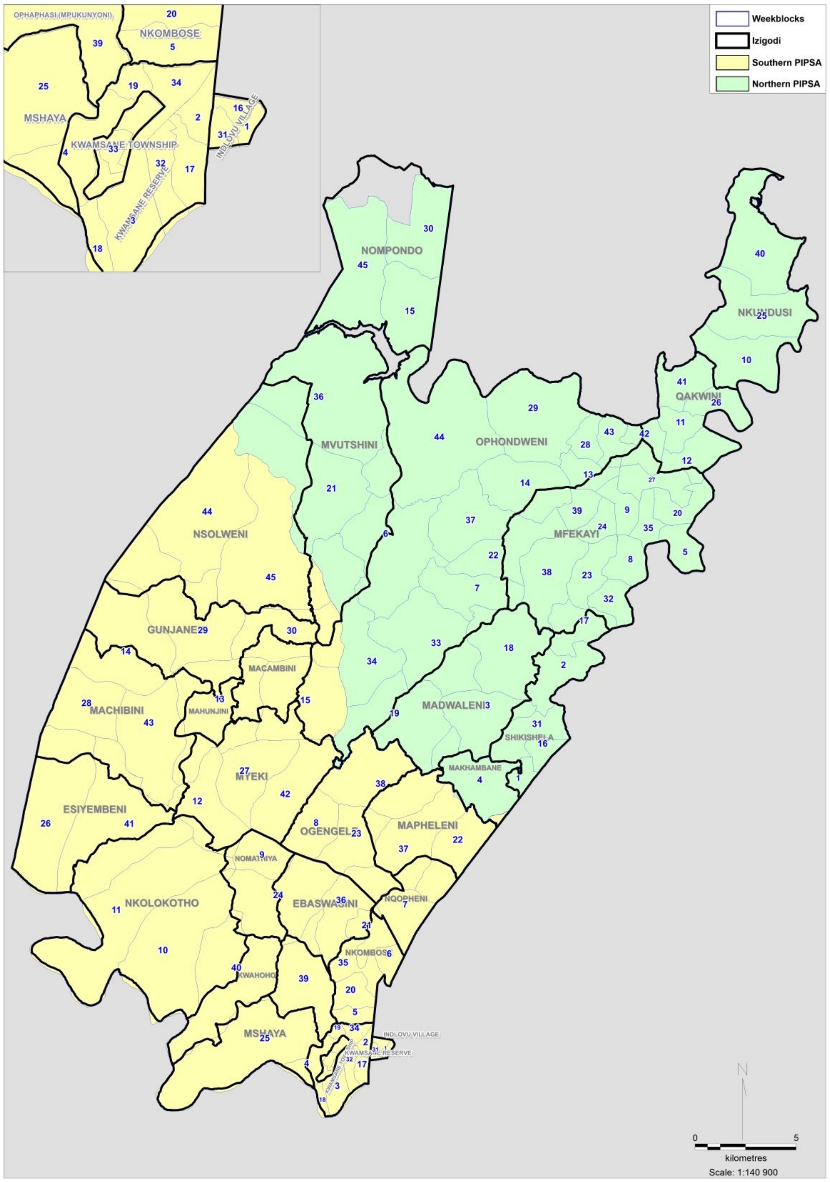

Figure 2_PIPSA_300

100x142mm (300 x 300 DPI)

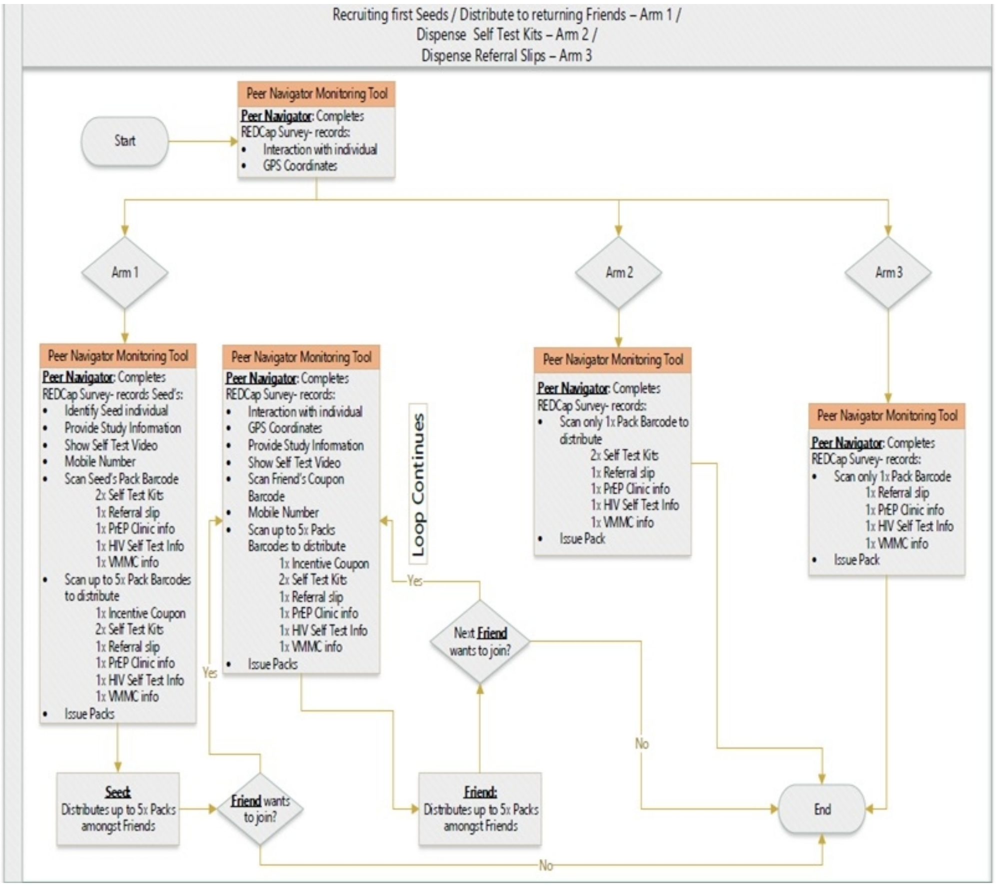

Fig3PeerNav_Process_300

68x59mm (300 x 300 DPI)

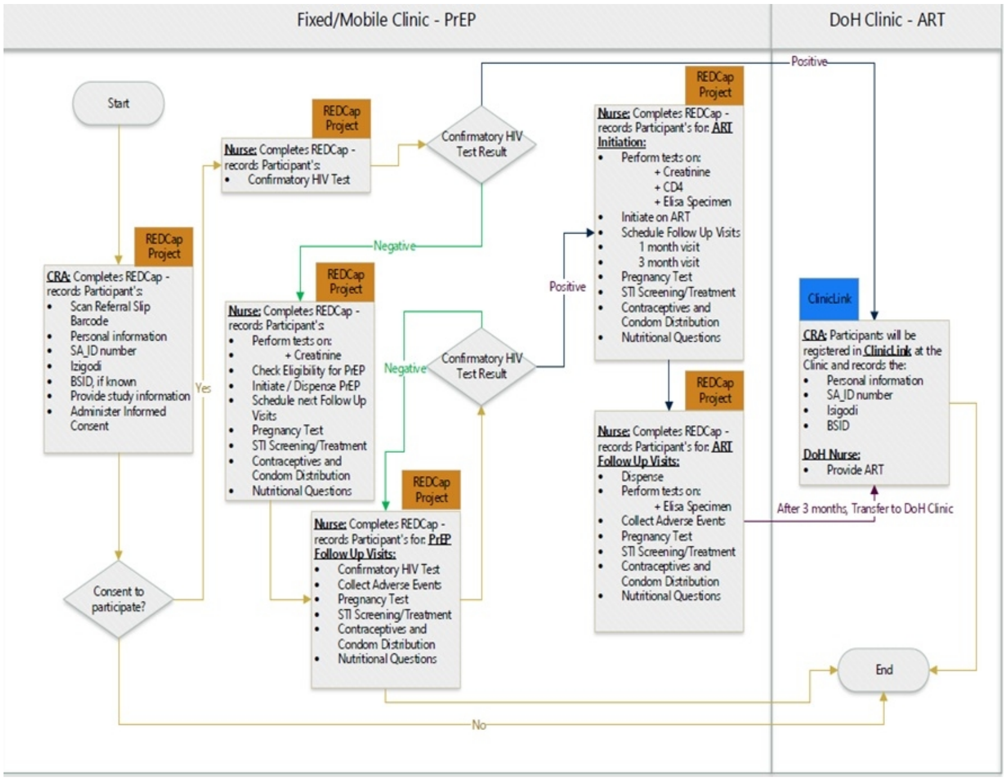

Fig4Mobileclinic_Process_300

75x58mm (300 x 300 DPI)

Reporting checklist for protocol of a clinical trial.

Based on the SPIRIT guidelines.

Instructions to authors

Complete this checklist by entering the page numbers from your manuscript where readers will find each of the items listed below.

Your article may not currently address all the items on the checklist. Please modify your text to include the missing information. If you are certain that an item does not apply, please write "n/a" and provide a short explanation.

Upload your completed checklist as an extra file when you submit to a journal.

In your methods section, say that you used the SPIRIT reporting guidelines, and cite them as:

Chan A-W, Tetzlaff JM, Altman DG, Laupacis A, Gøtzsche PC, Krleža-Jerić K, Hróbjartsson A, Mann H, Dickersin K, Berlin J, Doré C, Parulekar W, Summerskill W, Groves T, Schulz K, Sox H, Rockhold FW, Rennie D, Moher D. SPIRIT 2013 Statement: Defining standard protocol items for clinical trials. Ann Intern Med. 2013;158(3):200-207

		Reporting Item	Page Number
**Administrative information**			
Title	#1	Descriptive title identifying the study design, population, interventions, and, if applicable, trial acronym	1

For peer review only

Trial registration	#2a	Trial identifier and registry name. If not yet registered, name of intended registry	2
`	#2b	All items from the World Health Organization Trial Registration Data Set	(see below for specific items)
Protocol version	#3	Date and version identifier	2
Funding	#4	Sources and types of financial, material, and other support	17
Roles and responsibilities: contributorship	#5a	Names, affiliations, and roles of protocol contributors	1, 17
Roles and responsibilities: sponsor contact information	#5b	Name and contact information for the trial sponsor	17
Roles and responsibilities: sponsor and funder	#5c	Role of study sponsor and funders, if any, in study design; collection, management, analysis, and interpretation of data; writing of the report; and the decision to submit the report for publication, including whether they will have ultimate authority over any of these activities	17
Roles and responsibilities: committees	#5d	Composition, roles, and responsibilities of the coordinating centre, steering committee, endpoint adjudication committee, data management team,	N/A

For peer review only

		and other individuals or groups overseeing the trial, if applicable (see Item 21a for data monitoring committee)	
**Introduction**			
Background and rationale	#6a	Description of research question and justification for undertaking the trial, including summary of relevant studies (published and unpublished) examining benefits and harms for each intervention	2-3
Background and rationale: choice of comparators	#6b	Explanation for choice of comparators	N/A
Objectives	#7	Specific objectives or hypotheses	4
Trial design	#8	Description of trial design including type of trial (eg, parallel group, crossover, factorial, single group), allocation ratio, and framework (eg, superiority, equivalence, non-inferiority, exploratory)	4-5
**Methods: Participants, interventions, and outcomes**			
Study setting	#9	Description of study settings (eg, community clinic, academic hospital) and list of countries where data	6

For peer review only

		will be collected. Reference to where list of study sites can be obtained	
Eligibility criteria	#10	Inclusion and exclusion criteria for participants. If applicable, eligibility criteria for study centres and individuals who will perform the interventions (eg, surgeons, psychotherapists)	8-9
Interventions: description	#11a	Interventions for each group with sufficient detail to allow replication, including how and when they will be administered	5-6
Interventions: modifications	#11b	Criteria for discontinuing or modifying allocated interventions for a given trial participant (eg, drug dose change in response to harms, participant request, or improving / worsening disease)	N/A
Interventions: adherence	#11c	Strategies to improve adherence to intervention protocols, and any procedures for monitoring adherence (eg, drug tablet return; laboratory tests)	N/A
Interventions: concomitant care	#11d	Relevant concomitant care and interventions that are permitted or prohibited during the trial	N/A
Outcomes	#12	Primary, secondary, and other outcomes, including the specific measurement variable (eg, systolic blood pressure), analysis metric (eg, change from baseline, final value, time to event), method of aggregation (eg, median, proportion), and time point for each outcome. Explanation of the clinical	10-11

For peer review only

		relevance of chosen efficacy and harm outcomes is strongly recommended	
Participant timeline	#13	Time schedule of enrolment, interventions (including any run-ins and washouts), assessments, and visits for participants. A schematic diagram is highly recommended (see Figure)	7-8
Sample size	#14	Estimated number of participants needed to achieve study objectives and how it was determined, including clinical and statistical assumptions supporting any sample size calculations	10
Recruitment	#15	Strategies for achieving adequate participant enrolment to reach target sample size	7-8
**Methods: Assignment of interventions (for controlled trials)**			
Allocation: sequence generation	#16a	Method of generating the allocation sequence (eg, computer-generated random numbers), and list of any factors for stratification. To reduce predictability of a random sequence, details of any planned restriction (eg, blocking) should be provided in a separate document that is	9

For peer review only

		unavailable to those who enrol participants or assign interventions	
Allocation concealment mechanism	#16b	Mechanism of implementing the allocation sequence (eg, central telephone; sequentially numbered, opaque, sealed envelopes), describing any steps to conceal the sequence until interventions are assigned	9
Allocation: implementation	#16c	Who will generate the allocation sequence, who will enrol participants, and who will assign participants to interventions	9
Blinding (masking)	#17a	Who will be blinded after assignment to interventions (eg, trial participants, care providers, outcome assessors, data analysts), and how	9
Blinding (masking): emergency unblinding	#17b	If blinded, circumstances under which unblinding is permissible, and procedure for revealing a participant's allocated intervention during the trial	N/A
**Methods: Data collection, management, and analysis**			
Data collection plan	#18a	Plans for assessment and collection of outcome, baseline, and other trial data, including any related processes to promote data quality (eg, duplicate measurements, training of assessors) and a	11-12

For peer review only

		description of study instruments (eg, questionnaires, laboratory tests) along with their reliability and validity, if known. Reference to where data collection forms can be found, if not in the protocol	
Data collection plan: retention	#18b	Plans to promote participant retention and complete follow-up, including list of any outcome data to be collected for participants who discontinue or deviate from intervention protocols	N/A
Data management	#19	Plans for data entry, coding, security, and storage, including any related processes to promote data quality (eg, double data entry; range checks for data values). Reference to where details of data management procedures can be found, if not in the protocol	13
Statistics: outcomes	#20a	Statistical methods for analysing primary and secondary outcomes. Reference to where other details of the statistical analysis plan can be found, if not in the protocol	13
Statistics: additional analyses	#20b	Methods for any additional analyses (eg, subgroup and adjusted analyses)	13
Statistics: analysis population and missing data	#20c	Definition of analysis population relating to protocol non-adherence (eg, as randomised analysis), and	13

For peer review only

		any statistical methods to handle missing data (eg, multiple imputation)	
**Methods: Monitoring**			
Data monitoring: formal committee	#21a	Composition of data monitoring committee (DMC); summary of its role and reporting structure; statement of whether it is independent from the sponsor and competing interests; and reference to where further details about its charter can be found, if not in the protocol. Alternatively, an explanation of why a DMC is not needed	6
Data monitoring: interim analysis	#21b	Description of any interim analyses and stopping guidelines, including who will have access to these interim results and make the final decision to terminate the trial	6
Harms	#22	Plans for collecting, assessing, reporting, and managing solicited and spontaneously reported adverse events and other unintended effects of trial interventions or trial conduct	12
Auditing	#23	Frequency and procedures for auditing trial conduct, if any, and whether the process will be independent from investigators and the sponsor	N/A
**Ethics and dissemination**			

For peer review only

Research ethics approval	#24	Plans for seeking research ethics committee / institutional review board (REC / IRB) approval	16
Protocol amendments	#25	Plans for communicating important protocol modifications (eg, changes to eligibility criteria, outcomes, analyses) to relevant parties (eg, investigators, REC / IRBs, trial participants, trial registries, journals, regulators)	N/A
Consent or assent	#26a	Who will obtain informed consent or assent from potential trial participants or authorised surrogates, and how (see Item 32)	14
Consent or assent: ancillary studies	#26b	Additional consent provisions for collection and use of participant data and biological specimens in ancillary studies, if applicable	N/A
Confidentiality	#27	How personal information about potential and enrolled participants will be collected, shared, and maintained in order to protect confidentiality before, during, and after the trial	14
Declaration of interests	#28	Financial and other competing interests for principal investigators for the overall trial and each study site	16
Data access	#29	Statement of who will have access to the final trial dataset, and disclosure of contractual agreements that limit such access for investigators	16

For peer review only

Ancillary and post trial care	#30	Provisions, if any, for ancillary and post-trial care, and for compensation to those who suffer harm from trial participation	N/A
Dissemination policy: trial results	#31a	Plans for investigators and sponsor to communicate trial results to participants, healthcare professionals, the public, and other relevant groups (eg, via publication, reporting in results databases, or other data sharing arrangements), including any publication restrictions	14
Dissemination policy: authorship	#31b	Authorship eligibility guidelines and any intended use of professional writers	N/A
Dissemination policy: reproducible research	#31c	Plans, if any, for granting public access to the full protocol, participant-level dataset, and statistical code	N/A

Appendices

Informed consent materials	#32	Model consent form and other related documentation given to participants and authorised surrogates	See Supplementary file
Biological specimens	#33	Plans for collection, laboratory evaluation, and storage of biological specimens for genetic or molecular analysis in the current trial and for future use in ancillary studies, if applicable	N/A

For peer review only

None The SPIRIT checklist is distributed under the terms of the Creative Commons Attribution License CC-BY-ND 3.0. This checklist can be completed online using https://www.goodreports.org/, a tool made by the EQUATOR Network in collaboration with Penelope.ai

For peer review only

BMJ Open

A cluster randomised controlled trial to determine the effect of peer delivery HIV Self-Testing to support linkage to HIV prevention among young women in rural KwaZulu-Natal, South Africa: study protocol

Journal:	*BMJ Open*
Manuscript ID	bmjopen-2019-033435.R1
Article Type:	Protocol
Date Submitted by the Author:	06-Nov-2019
Complete List of Authors:	Adeagbo, Oluwafemi; Africa Centre for Health and Population Studies; University of Johannesburg, Sociology<br>Mthiyane, Nondumiso; Africa Centre for Health and Population Studies<br>Herbst, Carina; Africa Centre for Health and Population Studies<br>Mee, Paul; London School of Hygiene and Tropical Medicine,<br>Neuman, Melissa; London School of Hygiene and Tropical Medicine,<br>Dreyer, Jaco; Africa Centre for Health and Population Studies<br>Chimbindi, Natsayi; Africa Centre for Health and Population Studies<br>Smit, Theresa; Africa Centre for Health and Population Studies<br>Okesola, Nonhlanhla; Africa Centre for Health and Population Studies<br>Johnson, Cheryl; World Health Organization, Department of HIV/AIDS<br>Hatzold, Karin; Population Services International<br>Seeley, Janet; Africa Centre for Health and Population Studies; London School of Hygiene and Tropical Medicine,<br>Cowan, F; Liverpool School of Tropical Medicine, International Public Health; CeSHHAR Zimbabwe,<br>Corbett, Liz; LSHTM, Infectious and Tropical Diseases<br>Shahmanesh, Maryam; University College London, Institute for Global Health
**Primary Subject Heading**:	HIV/AIDS
Secondary Subject Heading:	Health services research, HIV/AIDS, Global health, Evidence based practice, Health policy
Keywords:	HIV/AIDS, Peer delivery model, South Africa, PrEP, ART, HIV Self-testing

SCHOLARONE™
Manuscripts

A cluster randomised controlled trial to determine the effect of peer delivery HIV Self-Testing to support linkage to HIV prevention among young women in rural KwaZulu-Natal, South Africa: study protocol

Adeagbo OA [1,2,4], Mthiyane TN[1], Herbst C[1], Mee P[3], Neuman M[3], Dreyer J[1], Chimbindi N[1,2], Smit T[1]; Okesola N[1], Johnson C[7], HatZold K[8], Seeley J[1,3], Cowan F[5,6], Corbett L[3], Shahmanesh M[1,2]

[1]Africa Health Research Institute, South Africa (formerly known as Africa Centre for Health & Population Studies)

[2]University College London, UK

[3]London School of Hygiene & Tropical Medicine, UK

[4]University of Johannesburg, South Africa

[5] The Centre for Sexual Health and HIV/AIDS Research, Zimbabwe

[6]Liverpool School of Tropical Medicine

[7]World Health Organisation, Switzerland

[8]Population Services International

Corresponding Author: Maryam Shahmanesh, Institute of Global Health, University College London, W61E JB, United Kingdom m.shahmanesh@ucl.ac.uk

ABSTRACT

Introduction:

A cluster randomised controlled trial (cRCT) to determine whether HIV self-testing (HIVST) delivered by peers either directly or through incentivised peer-networks, could increase the uptake of antiretroviral therapy and Pre-Exposure Prophylaxis amongst young women (18-24 years) is being undertaken in an HIV hyperendemic area in KwaZulu-Natal, South Africa.

Methods and analysis:

A 3-arm cRCT started mid-March 2019, in 24 areas in rural KwaZulu-Natal. Twenty-four pairs of peer navigators working with ~12000 young people aged 18-30 years over a period of 6 months were randomised to: (1) *incentivised-peer-networks (IPN):* peer-navigators recruited participants "seeds" to distribute up to 5 HIVST packs and HIV prevention information to peers within their social networks. Seeds receive an incentive (20 Rand = $1.5) for each respondent who contacts a peer-navigator for additional HIVST packs to distribute;(2) *peer-navigator-distribution (PND):* peer-navigators distribute HIVST packs and information directly to young people; (3) *standard of care (SOC):* peer-navigators distribute referral slips and information. All arms promote sexual health information and provide barcoded clinic referral

For peer review only - http://bmjopen.bmj.com/site/about/guidelines.xhtml

slips to facilitate linkage to HIV testing, prevention and care services. The primary outcome is the difference in linkage rate between arms, defined as the number of women (18-24 years) per peer-navigators month of outreach work (/pnm) who linked to clinic-based PrEP eligibility screening or started ART, based on HIV-status, within 90 days of receiving the clinic referral slip.

Ethics and dissemination:

This study was approved by the Institutional Review Boards at the World Health Organization, Switzerland (**Protocol ID: STAR CRT, South Africa**), London School of Hygiene and Tropical Medicine, UK (**Reference: 15990-1**), University of KwaZulu-Natal (**BFC311/18**), and the KwaZulu-Natal Department of Health (**Reference: KZ_201901_012**), South Africa. The findings of this trial will be disseminated at local, regional and international meetings and through peer-reviewed publications.

Trial registration number: Clinicaltrials.gov - NCT03751826. Protocol date: 17 January 2019

Keywords: HIV/AIDS; HIV Self-testing; peer delivery model; PrEP; ART; South Africa

XXXXXXXXXXXXXXXXXXXXXXXXXXXXXXXXXXXXXXXXXXXXXXXXXXXXXXXXXXXXXXXXXXXXXX

Strengths and limitations of this study

- There is no evidence on the use of HIVST to improve linkage to effective HIV prevention such as Pre-Exposure Prevention.
- There is limited evidence on the strength and limitations of different peer-to-peer approaches to improve uptake of HIV testing and linkage to care and prevention.
- Strengths include the use of a cRCT with rigorous measurement of the outcome linkage to care or prevention by arm, combined with process evaluation and cost-effectiveness studies.
- By embedding this cRCT within a longitudinal demographic surveillance setting, we are able to measure the population reach of the intervention.
- Limitations include a small risk of contamination across clusters and potential for coercive test or intimate partner violence.

XXXXXXXXXXXXXXXXXXXXXXXXXXXXXXXXXXXXXXXXXXXXXXXXXXXXXXXXXXXXXXXXXXXXXXX

INTRODUCTION

South Africa has the largest burden of HIV globally with 14% national prevalence rate and an estimated 7.9 million people living with HIV in 2017 [1]. The province of KwaZulu-Natal (KZN) is mostly affected by the epidemic with an 18.1% prevalence rate in 2017[1], while our research setting in uMkhanyakude district has an estimated 30% in the general population [2]. Of the new 88,000 HIV infections recorded amongst young people aged 15-24 years in 2017, 66 000 were among females[1]. Similarly, there is high HIV incidence rate in adolescent girls and young women (AGYW) with an estimated 5% per annum in aged 15-19 years and 8% per annum in aged 20-24 years respectively in our research setting in Hlabisa sub-district [3]. This high

For peer review only - http://bmjopen.bmj.com/site/about/guidelines.xhtml

incidence persists despite an increasing range of effective HIV prevention and treatment interventions, including condoms, antiretroviral (ART) based prevention e.g. pre-exposure prophylaxis (PrEP), universal test and treat (UTT) [4,5] and voluntary medical male circumcision (VMMC).

Evidence from South Africa and other countries in sub-Saharan Africa shows that this is partly due to the fact that many young people living with HIV are undiagnosed and therefore not linked to care [6,7]. Similarly, a recent treatment-as-prevention (TasP) trial conducted in this area failed to show an impact on incidence in part due to the challenge of testing and linking young women [8]. Patient level fears (e.g. stigma, labelling and discrimination) and facility level barriers (e.g. distance, waiting times and provider attitudes) continue to be barriers to young people not seeking HIV care services (HIV testing and uptake and adherence to antiretroviral therapy for treatment) in health facilities [9-11]. There is an urgent need to increase the proportion of those (particularly AGYW) who know their HIV status and take up effective HIV treatment as well as prevention – including ART based care and prevention.

To increase global testing rates and early access to treatment or PrEP, HIV self-testing (HIVST) – a simple saliva or blood-based self-test similar to a pregnancy test - has been identified as a potential method given its privacy and convenience [12-16]. Studies from different countries including South Africa have shown high acceptability and uptake of HIVST particularly amongst first time testers and young people [13,14,16-19]. Also, a growing number of studies have shown that rapid oral fluid testing was preferred to blood-based testing [20-22]. The OraQuick® In-home HIV test (OraSure Technologies, Inc., Bethlehem, PA, manufactured in Thailand) was recently pre-qualified by the World Health Organization for international procurement[23]. Field based use confirmed the high accuracy of HIVST, albeit with some variability across different educational levels[14,18,24]. HIVST (OraQuick) product is currently available in South Africa and has been endorsed by the National Department of Health (NDoH) in those aged 18 and above, with recommendations emphasizing the need for health care worker supported testing in those aged 18 and under.

Effective biomedical innovations such as PrEP have the potential to be gamechangers in the HIV epidemic in South Africa as part of the combination HIV prevention strategy and have thus been recommended for key populations such as sex workers, men who have sex with men and adolescent girls and young women aged 15-24 by the NDoH in South Africa[25]. However, their effectiveness will depend on HIV testing uptake and subsequent linkage to care and prevention [26-28]. The key findings from systematic reviews of the HIV treatment cascade suggest that: (1) community-based delivery models, including adherence clubs, community health workers delivering de-centralised care, and task-shifting to lay caregivers providing support across conditions, improve both ART uptake and sustained retention in low and middle-income settings [29-31]; (2) peer support is effective to deliver health intervention particularly to hard-to-reach groups [32,33]. Moreover, there is some evidence to suggest that HIVST can improve linkage to treatment when coupled with community based support [19,34]. However, there is limited evidence of the effectiveness of HIVST to link people who are negative to prevention, and in particular PrEP, with or without community-based support.

Here, we describe a cluster randomised controlled trial to address this critical gap in HIVST evidence and linkage for young women aged 18-24 years. Although all young people aged 18-30 years are included in the peer-led community based promotion of HIV testing and linkage to HIV prevention and care, the aim of this trial is to determine whether HIVST delivered by peers either directly or through incentivised peer-

For peer review only - http://bmjopen.bmj.com/site/about/guidelines.xhtml

networks, can increase the uptake of antiretroviral therapy (ART) and Pre-Exposure Prophylaxis (PrEP) amongst adolescent girls and young women (18-24 years) in a high HIV transmission setting in KwaZulu-Natal (KZN), South Africa. To the best of our knowledge, this is one of the first trials to test the effectiveness of oral-based HIVST to improve uptake of prevention in South Africa.

STUDY AIMS AND OBJECTIVES

The specific aims of this trial are to: (1) increase the knowledge of HIV status among young women aged 18-24 years old and their young male partners through the distribution of HIVST through incentivised peer networks or direct distribution by peer navigators compared to peer navigators referring them into HIV testing services; (2) determine an increase in the rate of linkage among young women aged 18-24 years to HIV prevention and treatment services facilitated by distribution of HIVST through incentivised peer networks or direct distribution by peer navigators compared to peer navigators referring into services; (3) determine an overall increase in young men and women aged 18-30 aware of their status and linked to HIV care and prevention; (4) conduct a process evaluation of the acceptability, feasibility, and reach (out of school, recently migrant and living in remote areas) in linking 18-24-year-old women to HIV prevention and treatment services of HIVST distribution through incentivised peer networks, or direct distribution by peer navigators or peer navigators referring into services and (5) measure the cost per 18-24-year-old woman linked to prevention and care through peer-led incentivised HIVST delivery system or direct distribution of HIVST by peer navigators, compared to peer navigator referring them to services.

METHODS/DESIGN

This is a 3-arm cluster randomised controlled trial (two intervention arms and one control arm) launched in mid-March 2019 and currently being carried out by 24 pairs of peer navigators that have been randomly assigned to one of three arms. The following sub-headings grapple with the methods, outcomes, procedures and study design amongst others.

Study setting and population

This study will be conducted in Africa Health Research Institute's (AHRI) long-standing demographic surveillance area in northern KwaZulu-Natal. The study area is mostly rural, and poor compared with other parts of South Africa, with high levels of unemployment (over 85% of young adults aged 20-24 years are unemployed) and the local language is IsiZulu[8]. In the study area, 8 out of 100 women aged between 20 and 24 years acquire HIV in one year, and 4 out of 10 women attending antenatal clinics are found to be infected with HIV. Data between 2011 and 2015 in the study area suggests that sexually active women aged 16-29 and young adult men have an HIV incidence above the threshold of eligibility for PrEP.

The demographic surveillance area provides over 16 years of household history, and over a million person-years of follow-up through annual individual-level surveys, which capture sexual behaviour and partnerships, reproductive histories and contraception use, access to HIV testing and care, access to HIV prevention services (including VMMC), as well as socio-demographic information. Moreover, through a Memorandum of Understanding with the Department of Health, AHRI has also embedded data collection clerks within the public health clinics to capture electronically any clinical attendance and linking it with

For peer review only - http://bmjopen.bmj.com/site/about/guidelines.xhtml

the surveillance platform on all consenting attendees. This allows us to measure linkage of individuals to HIV care and use of contraceptive services. As part of a NIH R01 we have selected, trained and employed 24 pairs of peer navigators, working in 24 discrete areas (based on administrative divisions) of the demographic surveillance area (the Hlabisa district of uMkhanyakude district of northern KwaZulu-Natal (KZN), South Africa) to deliver HIV and sexual health related Health promotion to an estimated 12000 youth (male and female) aged 18-30 years (~500 per each of the 24 administrative areas) and young women aged 18-24 years residing in the administrative areas (figure 1).

Figure 1: Map of study sites in Hlabisa sub-district in KwaZulu-Natal, South Africa

Theory of change

The intervention that is being tested in this cRCT is guided by a theory of change developed through mental models and deductive development[35] entrenched in ecological approach[36]. We theorised that the distribution of HIVST kits (including linkage information and referral slips) via peer navigators or peer social networks (respondent driven sampling - RDS) would lead to improved HIV prevention cascade, HIV testing uptake and linkage to HIV treatment or prevention services such as PrEP, amongst young women aged 18-24 years by creating peer-led demand, supporting young people to explore their candidacy for HIV care and prevention in privacy, and using social networks to reach those who need it most[37]. Work done by our group suggested that various factors associated with 'ecological framework' such as the fear of HIV-related stigma of attending a clinic for HIV testing and discrimination from healthcare providers or community may be addressed by HIVST since individuals can test privately anywhere without fear of being seen or judged[2,38]. Furthermore, formative work from our group suggested that community based delivery of services through youth friendly and accessible clinics for the study participants (walk-ins and those who present the study referral slips) could provide confirmatory HIV testing, treatment, prevention, contraceptives and other health services[39,40]. Following this, we developed a peer-to-peer intervention to reduce the burden of HIV among young women. We used the SPIRIT reporting guidelines in this article[41].

Trial design

This cRCT is comparing two models of peer delivery of HIVST in the study sites through incentivised respondent driven peer networks and direct distribution by peer navigators compared to standard of care (referral to HIV testing, prevention and care services by peer navigators) in improving the uptake of HIV testing, prevention and care amongst young women (18-24 years). Eight (8) pairs of peer navigators were randomised and assigned to each study arm with the intention of reaching young women aged 18-24 years with HIVST packs (including referral slips) and/or linkage information (including PrEP, contraceptives, ART etc.) during the six-month of community outreach. Peer navigators are randomised to 1 of 3 arms: 1) *incentivised-peer-networks:* peer-navigators recruited participants "seeds" to distribute up to 5 HIVST packs (including incentivised coupons) and HIV prevention information to peers within social networks. Seeds receive an incentive (20 Rand = $1.5) for each respondent who contacts a peer-navigator

For peer review only - http://bmjopen.bmj.com/site/about/guidelines.xhtml

for additional HIVST packs to distribute; (2) *peer-navigator-distribution:* peer-navigators distribute HIVST packs and information directly to young people; (3) *standard of care:* peer-navigators distribute referral slips and information. All arms promote sexual health and HIV care and prevention (including PrEP and ART) and provide barcoded clinic referral slips to facilitate linkage to HIV testing, prevention and care services (figure 2).

The unit of randomisation is the pair of peer navigators working in each of the 24 areas included in the study. The areas are not adjoining, and each is bordered by a natural boundary (e.g. roads or streams) or by a sizeable distance. Although contamination is inevitable in this type of cRCT, the spillover effects are contained by measuring the outcome by exposure to the peer-navigator cluster in multiple ways, including barcoded and colour coded referral slips as well as peer-navigator and ward names that determine participant exposure to specific intervention components. Coupled with this, we are conducting a mixed method process evaluation that provides context and add nuance to our understanding of any contamination.

Outcomes

The long-term goal of the intervention is to increase knowledge of HIV status and improve linkage to HIV care or prevention services such as PrEP amongst young women aged 18-24 years. A number of primary and secondary measures have been defined a priori. An interim analysis of the primary outcome will be conducted at 3 months.

Primary outcome:

The primary outcome compares the difference in linkage rate between arms, defined as the number of women (18-24 years) per peer-navigator month of outreach work (/pnm) who linked to clinic-based PrEP eligibility screening or started ART, based on HIV-status, within 90 days of referral.

Secondary outcomes:

The following calculations are planned for the secondary outcomes:

- Comparison of the difference per study arm of the total number of linkages (AGYW aged 18-24) per 100 clinic referral slips distributed.
- Comparison of the difference per study arm of the total number of linkages in men and women aged 18-30 per peer navigator outreach month.
- The change in proportion of young people aged 18-24 years who are aware of HIVST and who have used HIVST over time.
- Comparison of the difference per study area in the proportion of 18-24-year olds who report knowledge of HIV status and uptake of ART, PrEP and voluntary medical male circumcision (VMMC) in the surveillance area.
- The proportion of hard-to-reach adolescent girls and young women (aged 18-24 years) linked to care in the three study arms.

Description of study arms

Intervention arm 1 – incentivised network distribution of HIVST: n=8 pairs of peer navigators are using RDS approach to distribute HIVST with health promotion and linkage information (e.g. clinic referral slips

For peer review only - http://bmjopen.bmj.com/site/about/guidelines.xhtml

and information about HIV and PrEP). Each pair of peer navigator recruits n=10, 18-24 years old female seeds from the participating communities. Each seed fills a brief *service recipient questionnaire* – self-filled on a tablet. Following which they receive verbal health promotion from the peer-navigator on the HIV prevention services available, the importance of sexual health, the benefits of HIV testing PrEP and ART, and a demonstration of HIVST. Seeds are asked to recruit AGYW aged 18-24 years preferentially but not exclusively and to avoid distribution of HIVST to those under the age of 18 or over the age of 30. All seeds are asked to complete a brief check of their understanding of the information provided to them, particularly information about not using HIVST if someone is on ART, the window period, the recommended support to those under 18 using HIVST, and the need for confirmatory testing.

As shown in fig. 3, individuals who return with one of the coupons to a peer navigator undergo the same procedure as the seeds as described above. They are also given up to 5 uniquely numbered incentivised recruitment coupons and HIVST kits to pass on. When coupons are returned, the original individual who handed out the coupon receives a sum of R20 ($1.5) in airtime per friend or peer who returns the coupon. This sum is a reimbursement for the time that they have spent in explaining and demonstrating the use of an HIVST and is not seen to be an undue incentive to coerce members of their social network to participate. There is no gender restriction of those recruited through the networks, however the primary outcome will be measured in young women aged 18-24 years only.

Intervention arm 2: peer navigator direct distribution of HIVST**:** n=8 pairs of peer navigators are distributing HIVST packs with health promotion and linkage information (e.g. clinic referral slips and information about HIV and PrEP) directly to young people aged 18-30 years over a period of six months. Each person contacted fills a brief *service recipient questionnaire* – self-filled on a tablet. Following which they receive verbal health promotion from the peer-navigator on the HIV prevention services available, the importance of sexual health, the benefits of HIV testing, PrEP and ART, and a demonstration of HIV self-screening. All participants are asked to complete a brief check of their understanding of the information provided to them, particularly information about the unreliability of HIVST if someone is on ART, the window period, the recommended support to those under 18 using HIVST, and the need for confirmatory testing..

Control - arm 3**:** n=8 pairs of peer navigators are currently distributing packs with health promotion and linkage information (e.g. clinic referral slips and information about HIV and PrEP) to encourage young people aged 18-30 years to test for HIV at clinics, and link to services/care. Each female aged 18-30 approached fills a brief service *recipient questionnaire* – self-filled on a tablet. Following which they receive verbal health promotion from the peer-navigator on the HIV prevention services available, the importance of sexual health, the benefits of HIV testing PrEP and ART.

Figure 2: Flow diagram of trial enrolment, randomisation and intervention arms

For peer review only - http://bmjopen.bmj.com/site/about/guidelines.xhtml

Study oversight

An independent scientific Technical Advisory Group (TAG) was formed by the Unitaid-funded HIV Self-Testing Africa initiative (STAR – a consortium of scientists conducting HIV self-testing related research in different countries) to monitor and supervise the progress of data collection, provide independent review of data collected during all cRCTs conducted under the STAR initiative, and assist investigators in disseminating results. TAG comprises members with expertise in HIV epidemiology, statistics, health economics, social science and AGYW. TAG will convene periodic meetings to review data and discuss any issues emanating from this trial.

Study inclusion and exclusion criteria

Complete inclusion and exclusion criteria are summarised in tables 1 to 3. There are criteria for different recruitment stages in the trial. Above all, participants must not be less than 18 and not more than 30 years old. They must provide informed consent, not currently on ART and must be living in the study sites.

Table 1: Inclusion and exclusion criteria for receiving the intervention, i.e. the recruitment by Peer Navigators and/or Seeds to receive HIVST packs or clinical referral slips

**Inclusion criteria**	**Exclusion criteria**
Participant must not be older than 30 years and younger than 18 years	Participants under 18 years or older than 30 years
Participant must agree to participate	Participant unwilling to participate
Both males and females can be included	None
Must not be known to be on ART – based on self-report	If on ART

Table 2: Inclusion and exclusion criteria for ascertaining the primary outcome

**Inclusion criteria**	**Exclusion criteria**
Participant must not be older than 24 years and younger than 18 years	Participants under 18 years or older than 24 years
Provide written informed consent	Participants not willing to consent or unable to provide informed consent
Females	Males
Must not be known to be currently on ART	Currently on ART

Table 3: Inclusion and exclusion criteria for ascertaining the secondary outcome

**Inclusion criteria**	**Exclusion criteria**
Participant must not be older than 30 years and younger than 18 years	Participants under 18 years or older than 30 years
Provide written informed consent	Participants not willing to consent or unable to provide informed consent
Must not be known to be currently on ART	Currently on ART

For peer review only - http://bmjopen.bmj.com/site/about/guidelines.xhtml

Study recruitment and procedures

Study recruitment: Peer navigators are a cadre of recently matriculated youth or college graduates aged 18-30 years (male and female) recruited from the research community through the local municipal and traditional leaders. Between 6/2018-9/2018 participants underwent a 20-week training programme (3 days a week) which covered, youth development, HIV and sexual health information, HIV counselling and testing, confidentiality, ethics, and research methods, study procedures and HIVST. Progress was evaluated using written and oral assessments to select 48 peer-navigators to work in pairs and implement the intervention in their areas. The peer navigator intervention mirrors the South African cadre of community caregivers.

Before the peer navigators distribute the colour coordinated HIVST packs (each arm has its designated colour such as yellow, blue and pink) with unique identifiers in the intervention arms 1 and 2 or information packs in arm 3, the packs are being scanned and study participants are provided with information about the study and fill a brief *service recipient questionnaire* to be completed within Research Electronic Data Capture (REDCap - developed by Vanderbilt University, USA)[42] on a tablet (figure 3). Data collected at this point includes the date of recruitment, and the ID of peer navigator who recruited them, their age and area of residence. Participant's name, ID (e.g. SA national number), and telephone or WhatsApp contact are optional. Those who are recruited through RDS will also be asked to provide data on their network size, barcode of the RDS coupons and the additional HIVST kits are scanned for further distribution.

Peer navigators spend ~ 30 minutes with each willing participant to explain the benefits of linking to care and prevention. Those explaining HIVST need 15-20 minutes extra and some young people require more time or more visits. This data is captured in REDCap for the purpose of process evaluation and costing. This data will be used in an aggregate way to understand the process and cost of the service delivery. Individualised data from the survey will only be used in those participants that consent to their clinical data being linked and used for research purposes. If a participant withdraws their consent at any time, their data will be deleted from the research data set.

Study enrolment: Both walk-ins and study participants aged 18-30 years are eligible for receiving services from the designated study clinics. However, only those who have been referred through one of the three arms are eligible for study enrollment. This is being conducted by trained clinical research assistants in the designated study mobile and fixed clinics (figure 4). Also, all young women aged 18-24 years coming to one of the 11 primary health care clinics (PHC) or the mobile clinics in the surveillance sites are being directed by our AHRI data collection clerks to our research nurses. In both settings, the clinical research assistants or the research nurses explain the study and screen the young person for eligibility using a brief eligibility screening questionnaire on REDCap on a tablet. This includes questions to ascertain eligibility as well as arm of the study. If available, the clinic referral slip with the barcode with the unique identifier is scanned. The brief screening questionnaire that has further simple questions to ascertain if they were referred through any of the arms, i.e. receiving any of the three colour coded packs/referral slips, or contact with a named peer navigator, or referral through peer network. Interested eligible participants then go through the process of informed consent. Participants provide written informed consent for enrollment into the study and specifically to use the information on their linkage to care as an outcome and to link their baseline questions.

For peer review only - http://bmjopen.bmj.com/site/about/guidelines.xhtml

Clinical procedures: Irrespective of whether they consent to the study, all eligible individuals attending clinics are offered confirmatory HIV testing, with two point of care tests and then blood sent to laboratories for ELISA testing (Genscreen™ ULTRA HIV Ag-Ab qualitative immunoassay [4th Generation] Biorad, Marnes-la-Coquette, France). Individuals who test HIV-negative receive counseling around the benefits of PrEP and HIV-positive individuals receive counselling around the benefits of immediate starting of ART. If they agree, they will undergo clinical screening for PrEP and ART. Screening includes Point of Care (POC) tests for creatinine (StatSensor-I create-test strips, Nova Biomedical UK) to assess renal function and Hepatitis B (Alere Determine™ HBsAg, Alere International Limited, Ballybrit Galway, Ireland; Architect i2000 analyzer, Abbott Diagnostics, Abbott Park, IL, USA), with vaccination offered to those who are negative, and sexual behavior questionnaire to assess eligibility.

If HIV positive, ART is started by the professional nurses in any of 11 PHCs in the study sites. Patients who are eligible for PrEP are started in the three PrEP providing clinics (the mobile vans or the fixed urban adolescent and youth friendly clinic). Persons who are not eligible for PrEP receive counselling and, as indicated, a clinic referral with the screening results. A professional nurse initiates PrEP or ART usually on the same day or within two weeks of the screening visit. The professional nurse provides PrEP counselling that includes (1) *sexual health promotion,* with an emphasis on tackling the multiple health-related behaviors that will affect fertility and sexual pleasure (STIs, mental health, alcohol, diet and exercise); (2) assessment of fertility desire and contraception counselling; (3) choice of contraception and condoms; (4) HIV-negative men are also counselled around the benefits of VMMC and referred accordingly.

The counsellors provide counselling on adherence and develop an individualised adherence plan with the offer of face-to-face or virtual (WhatsApp/ text based) adherence support. If the participant agrees to immediate PrEP initiation, s/he is issued with a month's supply of generic tenofovir disoproxil fumerate and emtricitabine (TDF/FTC). Baseline and follow-up bloods are taken and processed as per SA National Department of Health guidelines. The professional nurse registers the participant at the clinic (or updates the record if the participant is already registered) so that the participant's records are available should the participant seeks care there. Participants receive a phone call seven days after initiating PrEP to complete a standard symptom screen for adverse effects and be referred to clinic for care if necessary. Participants have a clinic appointment scheduled one month, after PrEP initiation, as per national guidelines; appointments for refills and monitoring will be quarterly thereafter through either the mobile clinic, or other community-based refill points. Neutral text message reminders are provided for participants who have access to private messaging and phone calls. Participants are able to reschedule their appointments by text message, WhatsApp or calling the clinical hotline. Contact information is provided for the clinics whom participants can contact at any time.

Fig 3: Peer Navigators community outreach workflow

Fig 4: Mobile/Fixed clinics service workflow

For peer review only - http://bmjopen.bmj.com/site/about/guidelines.xhtml

Randomisation

We defined cluster as a pair of peer navigator (PN) who live and work in one of the 24 administrative areas that were included in the trial. Peer navigators were pre-assigned to 24 areas before the randomisation process. Using data from our recent DREAMS (combination HIV prevention) Impact Evaluation study which collected data from a representative sample of young women residing in the study areas, a restricted randomisation was applied to get balanced covariates (location, HIV testing prevalence and uptake of DREAMS combination HIV prevention by adolescent girls and young women) across the 3 arms. We generated a random set of possible 100,000 allocation options. After applying all 3 restrictions, a total of 47, 924 possible allocations remained, and a random number was generated and assigned to each allocation option. The random numbers were ranked from lowest to highest and the allocation option with the first rank was then selected. A randomisation list with intervention arms named alphabetically (A, B & C) was generated.

Following the statistical randomisation, a public randomisation was conducted where peer navigators were divided into three groups (A, B & C). Each group had 16 allocated PNs and 3 floating ones. Each group chose a suitable name and a leader who represented them. Group leads picked a concealed number to determine the order of picking their study arm from a box. The facilitator shook the box so to make sure that each concealed arm in the box had an equal chance of being picked. Lastly, the leaders were asked to open and announce the arms of their respective groups to the bigger group.

Blinding

The statistician and clinical staff did not participate in the public randomisation with peer navigators and they will remain blinded until the results of the study have been finalised.

Sample size calculations

Based on 2017 data, we estimated ~500 age eligible 18-30 years olds live in each peer navigator team catchment area, of whom we anticipated at least 200, 18-24-year-old females will be handed a study pack (so cluster size at least 200). We estimated this based on 2 peer navigators working approximately 1000 hours over the study period per cluster. We estimated that they would reach 2 young adults per each 4 hour of work and at least one would accept a study pack. We calculated the sample size calculation using the primary outcome, the rate of linkage after 90 days among women ages 18-24 years. Using our existing data on uptake of HIV testing in the DREAMS interventions as well as our data on uptake of testing and linkage to HIV care in the demographic surveillance rounds of, we estimated that 1 woman would link per 6 months of peer navigators outreach work time in the standard of care. With 8 peer educator pairs (or clusters) per arm and a cluster coefficient of variation (k) of 0.25, we will have 80% power to detect a 100% increase in rate from 1 woman to 2 women per 6 months of follow-up, and 90% power to detect a 150% increase from 1 woman to 2.5 women per 6 months of follow-up. We have chosen policy and clinically relevant increases in linkage to care. Assuming additional clustering of the outcome within peer educators and increasing the coefficient of variation (k) to 0.35, we have 80% power to detect a 150% increase in rate from 1 woman to 2.5 women per 6 months of follow-up. All sample size calculations assume two-tailed statistical tests with alpha=0.05.

For peer review only - http://bmjopen.bmj.com/site/about/guidelines.xhtml

Process evaluation

Our aim is to assess the acceptability, feasibility, and fidelity of the peer delivery model in each arm in facilitating linkage to care. We compare the pattern of recruitment per arm and assess the proportion of hard-to-reach AGYW (aged 18-24 years) - defined as out of school, recently migrated and those who live in remote areas linked to care in the three study arms. We will also explore potential unintended consequences and ethical issues that arise during peer referral and HIVST and ascertain what works for whom and when to be able to modify the intervention to improve equitable reach and coverage. Specifically, we will explore the reach of network recruitment compared to peer outreach work, in terms of reaching more vulnerable groups (out of school, recently migrated, and those who live more remotely). Entrenched in realist evaluation, this process evaluation uses a mixed method approach (quantitative and qualitative research techniques) to investigate implementation, mechanisms of impact and contextual factors, informed by the United Kingdom medical research council (MRC) guide[43] and wider implementation science literature with a focus on fidelity, reach and acceptability [44].

Cost-effectiveness evaluation

We compare the costs in intervention and control arms. Cost per case linked to PrEP eligibility assessment (HIV-) and cost per case started on ART (HIV+). To establish costs, we are using both a bottom-up ingredient-based costing approach and a top-down costing approach using the study budgets and expenditure reports. Specifically, we calculate and cost the actual time spent by peer-navigators in each arm for each person linked to care and prevention.

Data Collection

Participant Survey and Clinic Linkage:

A short survey (service recipient questionnaire) is administered to consenting individuals participating in the study. The questionnaire collects data on participant's demographic information and coupon identification. The data is captured on REDCap on a tablet. The survey takes approximately 5 minutes to complete and is administered in both English and isiZulu. The primary outcome of linkage is measured through identifying the consenting eligible young women who link to care through the 11 PHCs and the mobile clinics. We use an algorithm to identify which arm the individual came from, including the barcode on the referral slip they bring, the colour of the referral slips or HIVST pack, their area of residence, and the identity of the peer-navigator that recruited them.

Programmatic Data:

In addition to the survey, we collect the programme data records from the peer navigators daily reporting of their outreach activities. This includes the number of young people they have counselled and the numbers they have referred to services and the brief service recipient data they have collected on those who have received referral slips or HIVST packs. We use the programme data in an aggregate way (disaggregated only by gender) to understand the reach and coverage of the programme and compare that with those who link to care. We also use data on changes in self-reported HIVST and linkage services collected through the population intervention surveillance platform.

For peer review only - http://bmjopen.bmj.com/site/about/guidelines.xhtml

Participant in-depth interviews (IDIs):

IDIs are being conducted with the peer navigators (n= 30), clinical team (n= 6) stationed in clinics in the participating communities and a purposive sample of young women aged 18-24 years (including young men n= 45; 30 females[10 per arm], 15 males [5 per arm]) across the three arms and clinics. The interviews are conducted by trained social scientists fluent in English and isiZulu and take approximately 60 minutes in length depending on the participant's responses, and this enables the researchers to understand, contextualize and explore participants perceptions of the study and some of the issues emanating from the trial. The small number of IDI participants in qualitative study is allowed since deeper meanings of concepts and thematic areas are explored. To limit disturbances and ensure privacy, the IDIs are conducted in a private space suitable for the participant, and audio recorded with interviewees' consents. Prior to the interview, participants are encouraged to use pseudo names instead of their real names.

Statistical Analysis

The analysis of primary outcome follows an intention-to-treat (ITT) and per-protocol approaches. The analysis of secondary outcomes will be undertaken using per-protocol approach only. The primary outcome compares the difference between the rate of linkage of 18-24-year-old women to HIV confirmatory HIV testing, ART (if HIV positive) or PrEP counselling (if HIV negative). The rate is defined as the number of linkages per month of peer navigator outreach activities. The numerator is defined as the number of young women aged 18-24 who attend clinic for confirmatory HIV testing, PrEP counselling or ART, following HIV-ST distribution or peer navigator referral to HIV testing, treatment and prevention services. The denominator for intention-to-treat analysis (ITT) is the entire time (study duration) spent by peer navigators doing their peer outreach work. For the on-treatment analysis, we will use the actual time spent by peer navigators on distributing packs in each arm. The time worked by each peer navigator will be combined to get the total time per pair of peer navigator. The difference in rate of linkage between the study arms will be calculated - incentivised HIVST delivery through peer network and direct distribution of HIVST will be compared to standard of care.

Since we randomised the pairs of peer navigator (clusters), the rate of linkage will be calculated for each pair of peer navigator using aggregate data for each cluster. Since the number of clusters are small, the effect of the intervention will be estimated using a two-stage approach based on cluster-level summaries[45]. The cluster-level approach, although less statistically efficient than methods based on individual level regression, is more robust when there are a relatively small number of clusters. All analyses will be performed using STATA version 15 (StataCorp LP, College Station, Texas USA).

Cluster-level linkage rates will be calculated and used to estimate the unadjusted rate ratio and its 95% confidence interval for the effect of each intervention arm compared with the standard of care; the mean difference in linkage rates between each arm and standard of care, and against each-other will be assessed using a t-test. A rate ratio adjusting for substantial covariate imbalance at baseline will also be calculated, using a two-stage process; all covariates will be pre-specified in the analysis plan. To identify covariates for adjustment, baseline characteristics of each arm will be presented, and the size of the difference of covariates known to be associated with the outcome will be assessed quantitatively.

As part of the exploratory analysis, we will perform a (i) subgroup analysis by gender and area and (ii) two intervention arms will be compared to one another (incentivised HIVST delivery through peer network approach will also be compared to direct distribution of HIVST approach). To expand on this, the data

For peer review only - http://bmjopen.bmj.com/site/about/guidelines.xhtml

from the client survey captured on REDCap dashboard will be exported into STATA, cleaned and analysed. All reporting will conform to CONSORT guidance for cluster randomised trials[44,45].

Qualitative analysis

Nvivo software will be used for categorisation and coding of emerging themes from the interview transcripts. Identified themes (including participants' quotes) and interview transcripts will be reviewed and compared by the research team for inconsistencies and adequate representation of participants' views. Emerging themes that address the key focus of the study will be examined and analysed following an interpretivist approach [46].

Adverse events reporting

HIV testing, including HIVST, is well established and known to have a high level of safety. However, harmful reactions can occur. Adverse events (AE) related to HIVST include all undesirable experiences that result directly from use of the HIVST kit itself or as a reaction from others due to the presence of the kit, use of the kit or results produced from the kit. AEs can be from one person to another, or a person to themselves, and can occur before, during or after self-testing. We rely on participants to report any AEs to the study staff or through the hotline provided on the referral slip. Also, during PrEP resupply and monitoring visits, participants complete a standardised symptom screening questionnaire for adverse effects of PrEP as per South African clinical guidelines. Furthermore, all participants will receive regular creatinine tests to monitor their renal function. Participants who have severe (grade 3/4) adverse effects and serious adverse effects, are referred to the study clinician for medical evaluation. All participants who experience adverse events receive follow-up until the adverse event is resolved.

AEs and Serious Adverse Events (SAE) are captured through the process evaluation and community engagement units and the telephone hotline. In addition, peer navigators and clinic staff log AEs using our incident reporting form for to up to 12 months after the start of the intervention. Reported AEs and SAEs are monitored, categorised based on an established grading system. SAEs are logged, with the Principal Investigator to evaluate the SAE for seriousness and likely relationship to the intervention. Related SAEs are reported to UKZN and LSHTM Ethics Review Boards. All SAEs are reported regularly through six-month progress reports to TAG members, local and international collaborators. Annual reports with full listings of SAEs will be submitted to Ethics Review Boards.

Data management

Quantitative data are collected directly on the study tablet via REDCap database and resides within a single MySQL database server within a secure server cluster. Study-specific electronic laboratory results are transferred directly to a secured server for storage. Qualitative data are stored in the form of Word files or in Excel both of which can be uploaded into Nvivo qualitative data management programme. The use of MS Word will ensure that data can in future be shared for use in different analysis programmes.

For peer review only - http://bmjopen.bmj.com/site/about/guidelines.xhtml

These files will be kept on a secure access-controlled folder on a secured server. Qualitative audio files will be destroyed once they have been transcribed, translated and quality controlled.

Patient and public involvement

Although we did not involve patients or the public in the design of the study, findings from previous studies conducted within the community were useful during the study design phase. The study was also presented to the community advisory board (CAB) and the district department of health for comments before it was submitted to IRBs for ethics approval. The results of the study will be shared with the peer navigators and the research community through community dialogues and the community advisory board.

ETHICS AND DISSEMINATION

Ethical consideration: confidentiality and informed consent

All staff (including peer navigators) have been provided with training on research ethics such as confidentiality, voluntary participation and good clinical practices. Anonymity and confidentiality are ensured at all levels of the research process, and none of our reports, presentations or articles will contain study participants identifying information. Pseudonyms are used when reporting the data particularly qualitative data. Each participant is assigned a unique non-identifying participant identification number. Prior to their involvement in the study, participants are provided with adequate information about the study and are allowed to ask questions for clarifications. Voluntary informed consent is collected only if participants have the full understanding of the study procedures. A copy of the signed consent form is given to them. Participants are informed about the importance of the confirmatory diagnostic testing. An anonymous support hotline is provided on the referral slips should they need to discuss their HIV status, counselling and further linkage to HIV care or other health services. All participant irrespective of their consent to participating in the study are eligible for the clinical services provided through the study. The study was approved by and conforms to the ethical guidelines and standards of UKZN, LSHTM and WHO.

Dissemination plan

The results of this study will be disseminated through traditional academic channels (peer reviewed journal publications) as well as on different information dissemination platforms such as conferences, workshops, community meetings and symposia. The results of the study will also be presented at Self-Testing Africa (STAR) consortium meetings and will be included in the WHO guidelines. The results of the study will be shared with the peer navigators and the research community through community dialogues and the AHRI community advisory board. A detailed findings report will be shared with the Department of Health and other stakeholders to inform policy.

DISCUSSION

Despite the burden of HIV and the availability of free HIV testing and treatment in our local PHCs, HIV status knowledge remains low amongst young people < 30 years[2]. Several complex barriers (e.g. stigma, confidentiality, family rejection, waiting times and lack of youth friendly services etc.) impeding on young people's access to HIV care services were identified in a formative research we completed in 2018[2,38]. Studies including systematic reviews[13,19] have shown HIVST to be a promising alternative HIV testing

For peer review only - http://bmjopen.bmj.com/site/about/guidelines.xhtml

option because it is private, flexible and an efficient method. However, there is limited evidence on the use of HIVST to improve linkage to prevention such as PrEP. The overarching goal of this trial is to address the gap in HIV testing and linkage to HIV prevention among young women aged 18-24 years. This goal will be achieved through the assessment of two HIVST delivery models (incentivized peer network versus direct distribution by peer navigators) compared to the standard of care of peer navigator only.

A major strength of this study is the development of a theoretically derived intervention that can be implemented through existing cadre of community caregivers and peer to peer networks across SSA[47-50]. If found to be effective in increasing HIV testing uptake and prevention, the intervention is designed to be rolled out. Also, using a rigorous cost effectiveness analysis will allow SA policy makers to evaluate the cost-benefit ratio of using the different models of distribution in different settings. Furthermore, by collecting rigorous data on linkage to prevention both through the trial and from our surveillance infrastructure, we can understand the potential population impact of the different methods of HIVST distribution on knowledge of status and linkage to care and prevention. Ultimately this will provide evidence of the potential of the intervention to attract young people into the HIV care and prevention cascade and inform the evidence base to reduce the mortality and morbidity in youth. Lastly, rigorous process evaluation and collection of data on all adverse events and social harms will provide important data around some of the concerns about HIVST, i.e. the potential for coercive test, depression, anxiety, suicidality or intimate partner violence.

In order to reduce the risk of contamination due to the proximity of the areas and the nature of the intervention, we have piloted several methods including the use of referral slips with unique codes and colour coordinated packs to identify the arm individuals come from when they link to care. Previous data from nested cohorts that we have followed up in our area has shown that young people rarely migrate within our surveillance area given that only 220 of 2 184 young people < 25 years cohorts moved from one cluster to another in 2017/2018. Furthermore, the process evaluation will help us understand the delivery model in each arm as well as unintended consequences and ethical issues that arise during the study and ascertain what works for whom and when to be able to modify the intervention to improve equitable reach and coverage.

In conclusion, the results of this trial are expected to contribute to WHO guidelines and informed policy aimed at implementation and scale-up of HIVST and PrEP in South Africa. Also, this study will address critical gaps in the literature on HIV testing and prevention interventions for young people particularly females aged 18-24 years in Southern Africa.

Acknowledgements:

The authors acknowledge TAG of the STAR initiative and AHRI HIV Prevention Multilevel Group including the research assistants, peer navigators, clinical team, managers and research administrators for their commitment to the study. We also extend our appreciation to our research community including the community advisory boards in uMkhanyakude district.

For peer review only - http://bmjopen.bmj.com/site/about/guidelines.xhtml

Authors Contributions:

MS and LC conceived the study. OA, MS, CH, JD, JS, LC, FC, NC, NO, PM, MTN MN designed the study. OA and MS wrote the first draft of the manuscript. OA, MS, LC, JS, JD, CH, TS, FC, MN, NC, KH, CJ, PM, MTN, and NO read and critically revised the manuscript. All authors read and approved the final manuscript.

Funding:
This study is part of the Self-Testing Africa (STAR) initiative funded by the Unitaid (grant number: PO#10140-0-600). MS is partly supported by the US National Institute of Health (NIH) R01 (award no: 5R01MH114560-03) that supports a peer led outreach team of navigators to support uptake and retention of adolescents and young adults in existing HIV prevention. Africa Health Research Institute is supported by core funding from the Wellcome Trust [Core grant number (082384/Z/07/Z)]. The funders were not involved in the design of the study.

Competing interests:
None declared.

Ethics approval:

Ethical approval was obtained for the study from Institutional Review Boards (IRB) of the World Health Organisation, Switzerland (**Protocol ID: STAR CRT, South Africa**), London School of Hygiene and Tropical Medicine, UK (**Reference no: 15990 - 1**), University of KwaZulu-Natal (**BFC311/18**), and the KwaZulu-Natal Department of Health (**NHRD Reference no: KZ_201901_012**) , South Africa. The study was also approved by the community advisory board (CAB) representing the research community before it was submitted to IRBs. ***Trial registration number***: Clinicaltrials.gov - NCT03751826. Protocol date: 17 January 2019.

Data sharing statement:
On completion of the trial, de-identified data will be available through the Africa Health Research Institute, South Africa (AHRI) data access repository upon request. Requests for data sharing are reviewed by the data custodian and a Data Sharing committee at AHRI. After approval, all data sharing, even those without identifiers, will be through a secure and password-controlled data sharing site, with access strictly limited to transcripts that are relevant to the analysis planned. Users will be asked to abide by the AHRI Data Access and Sharing Policy.

REFERENCES

1. HSRC. *South African National HIV Prevalence, Incidence, Behaviour and Communication Survey 2017*

Pretoria, South Africa: Human Science Research Council July, 2018 2018.

2. Adeagbo O, Herbst C, Dlamini N, et al. The 'Missing Middle' in HIV Treatment Cascade: Ensuring Men's and Young People's access to HIV Care in KwaZulu-Natal, South Africa. Association of Social Science and Humanities in HIV , 2018 International AIDS Society Pre-conference 21 July 2018, 2018; Amsterdam.

For peer review only - http://bmjopen.bmj.com/site/about/guidelines.xhtml

3. M S, N C, N M, et al. Persistently high incidence of HIV in adolsescent girls and young women in rural KwaZulu-Natal, South Africa: Lessons for Determined Resilient, Empowered, AIDS free, Mentored and Safe (DREAMS) multilevel HIV prevention scale-up. Paper presented at: AIDS Impact; 17 November 2017, 2017; Cape Town, South Africa.
4. Organization WH. WHO expands recommendation on oral preexposure prophylaxis of HIV infection (PrEP): Policy brief. *WHO, Geneva.* 2015.
5. (NDoH) NDoHSA. *National Policy on HIV Pre Exposure Prophylaxis (PrEP) and test and treat (T&T).* Pretoria, South Africa2016.
6. Huerga H, Van Cutsem G, Farhat JB, et al. Progress towards the UNAIDS 90–90-90 goals by age and gender in a rural area of KwaZulu-Natal, South Africa: a household-based community cross-sectional survey. *BMC public health.* 2018;18(1):303.
7. Carrasco MA, Fleming P, Wagman J, Wong V. Toward 90-90-90: identifying those who have never been tested for HIV and differences by sex in Lesotho. *AIDS care.* 2018;30(3):284-288.
8. Iwuji C, Orne-Gliemann J, Larmarange J, et al. Universal test and treat and the HIV epidemic in rural South Africa: a phase 4, open-label, community cluster randomised trial. *The lancet HIV.* 2018;5(3):e116-e125.
9. Plazy M, Farouki KE, Iwuji C, et al. Access to HIV care in the context of universal test and treat: challenges within the ANRS 12249 TasP cluster-randomized trial in rural South Africa. *Journal of the International AIDS Society.* 2016;19(1):20913.
10. Moshabela M, Zuma T, Orne-Gliemann J, et al. “It is better to die”: experiences of traditional health practitioners within the HIV treatment as prevention trial communities in rural South Africa (ANRS 12249 TasP trial). *AIDS care.* 2016;28(sup3):24-32.
11. Boyer S, Iwuji C, Gosset A, et al. Factors associated with antiretroviral treatment initiation amongst HIV-positive individuals linked to care within a universal test and treat programme: early findings of the ANRS 12249 TasP trial in rural South Africa. *AIDS care.* 2016;28(sup3):39-51.
12. Spielberg F, Levine RO, Weaver M. Self-testing for HIV: a new option for HIV prevention? *The Lancet infectious diseases.* 2004;4(10):640-646.
13. Krause J, Subklew-Sehume F, Kenyon C, Colebunders R. Acceptability of HIV self-testing: a systematic literature review. *BMC public health.* 2013;13(1):735.
14. Choko AT, Desmond N, Webb EL, et al. The uptake and accuracy of oral kits for HIV self-testing in high HIV prevalence setting: a cross-sectional feasibility study in Blantyre, Malawi. *PLoS medicine.* 2011;8(10):e1001102.
15. Makusha T, Knight L, Taegtmeyer M, et al. HIV self-testing could “revolutionize testing in South Africa, but it has got to be done properly”: perceptions of key stakeholders. *PloS one.* 2015;10(3):e0122783.
16. Knight L, Makusha T, Lim J, Peck R, Taegtmeyer M, van Rooyen H. “I think it is right”: a qualitative exploration of the acceptability and desired future use of oral swab and finger-prick HIV self-tests by lay users in KwaZulu-Natal, South Africa. *BMC research notes.* 2017;10(1):486.
17. Spielberg F, Critchlow C, Vittinghoff E, et al. Home collection for frequent HIV testing: acceptability of oral fluids, dried blood spots and telephone results. *Aids.* 2000;14(12):1819-1828.
18. Belza MJ, Rosales-Statkus ME, Hoyos J, et al. Supervised blood-based self-sample collection and rapid test performance: a valuable alternative to the use of saliva by HIV testing programmes with no medical or nursing staff. *Sex Transm Infect.* 2012:sextrans-2011-050131.
19. Harichund C, Karim QA, Kunene P, Simelane S, Moshabela M. Exploring factors that influence the integration of HIVST with HCT using a qualitative comparative cross-over design in KwaZulu-Natal, South Africa. *Global public health.* 2019:1-13.
20. Gaydos CA, Hsieh Y-H, Harvey L, et al. Will patients “opt in” to perform their own rapid HIV test in the emergency department? *Annals of emergency medicine.* 2011;58(1):S74-S78.

For peer review only - http://bmjopen.bmj.com/site/about/guidelines.xhtml

21. Kalibala S, Tun W, Muraah W, Cherutich P, Oweya E, Oluoch P. Knowing myself first': feasibility of self-testing among health workers in Kenya. *Nairobi: Population Council.* 2011.
22. Lee VJ, Tan SC, Earnest A, Seong PS, Tan HH, Leo YS. User acceptability and feasibility of self-testing with HIV rapid tests. *JAIDS Journal of Acquired Immune Deficiency Syndromes.* 2007;45(4):449-453.
23. WHO. *Guidelines on HIV self-testing and partner notification.* Switzerland: WHO;2016.
24. Kapaku K, Neuman M, Maluzi K, et al. Is OraQuick HIV-self-testing valid among intended users? Analysis from a clinical performance study in Lusaka, Zambia. *International AIDS Society (IAS 2017) Paris, France.* 2017.
25. Cowan FM, Delany-Moretlwe S, Sanders EJ, et al. PrEP implementation research in Africa: what is new? *Journal of the International AIDS Society.* 2016;19:21101.
26. Van Damme L, Corneli A, Ahmed K, et al. Preexposure prophylaxis for HIV infection among African women. *New England Journal of Medicine.* 2012;367(5):411-422.
27. Marrazzo JM, Ramjee G, Richardson BA, et al. Tenofovir-based preexposure prophylaxis for HIV infection among African women. *New England Journal of Medicine.* 2015;372(6):509-518.
28. Yun K, Xu J-j, Zhang J, et al. Female and younger subjects have lower adherence in PrEP trials: a meta-analysis with implications for the uptake of PrEP service to prevent HIV. *Sex Transm Infect.* 2017:sextrans-2017-053217.
29. Kim S-H, Gerver SM, Fidler S, Ward H. Adherence to antiretroviral therapy in adolescents living with HIV: systematic review and meta-analysis. *AIDS (London, England).* 2014;28(13):1945.
30. Sharma M, Barnabas RV, Celum C. Community-based strategies to strengthen men's engagement in the HIV care cascade in sub-Saharan Africa. *PLoS medicine.* 2017;14(4):e1002262.
31. Van Rooyen H, Barnabas RV, Baeten JM, et al. High HIV testing uptake and linkage to care in a novel program of home_based HIV counseling and testing with facilitated referral in KwaZulu-Natal, South Africa. *Journal of acquired immune deficiency syndromes (1999).* 2013;64(1):e1.
32. Kanters S, Park JJ, Chan K, et al. Interventions to improve adherence to antiretroviral therapy: a systematic review and network meta-analysis. *The Lancet HIV.* 2017;4(1):e31-e40.
33. Pettifor A, Nguyen NL, Celum C, Cowan FM, Go V, Hightow-Weidman L. Tailored combination prevention packages and PrEP for young key populations. *Journal of the International AIDS Society.* 2015;18(2S1).
34. Choko AT, MacPherson P, Webb EL, et al. One Year Outcomes Following Community-Based HIV Self-Testing: A Prospective Study in Malawi. Conference on Retroviruses and Opportunistic Infections 2014; United States.
35. Funnell SC, Rogers PJ. *Purposeful program theory: Effective use of theories of change and logic models.* Vol 31: John Wiley & Sons; 2011.
36. Baral S, Logie CH, Grosso A, Wirtz AL, Beyrer C. Modified social ecological model: a tool to guide the assessment of the risks and risk contexts of HIV epidemics. *BMC public health.* 2013;13(1):482.
37. Hargreaves JR, Delany-Moretlwe S, Hallett TB, et al. The HIV prevention cascade: integrating theories of epidemiological, behavioural, and social science into programme design and monitoring. *Lancet HIV.* 2016;3(7):e318-322.
38. Adeagbo O, Carina H, Blandford A, et al. Building Mobile Phone-connected Diagnostics: A Qualitative Study Exploring People's 'Candidacy' for Mobile Health-Supported

HIV Testing and Care Services in Rural KwaZulu-Natal, South Africa. *Journal of medical Internet research.* 2019;21(9).
39. Adeagbo O, Herbst C, Dlamini N, et al. " If She Tests Negative, It Means I Am Also Negative": Men's Construction of HIV Testing in South Africa. *AIDS RESEARCH AND HUMAN RETROVIRUSES.* 2018;34:85-85.

For peer review only - http://bmjopen.bmj.com/site/about/guidelines.xhtml

40. Nkosi B, Seeley J, Ngwenya N, et al. Exploring adolescents and young people's candidacy for utilising health services in a rural district, South Africa. *BMC Health Services Research.* 2019;19(1):195.
41. Chan A-W, Tetzlaff JM, Altman DG, et al. SPIRIT 2013 statement: defining standard protocol items for clinical trials. *Annals of internal medicine.* 2013;158(3):200-207.
42. Harris PA, Taylor R, Thielke R, Payne J, Gonzalez N, Conde JG. Research electronic data capture (REDCap)—a metadata-driven methodology and workflow process for providing translational research informatics support. *Journal of biomedical informatics.* 2009;42(2):377-381.
43. Moore GF, Audrey S, Barker M, et al. Process evaluation of complex interventions: Medical Research Council guidance. *bmj.* 2015;350:h1258.
44. Jacobs C, Michelo C, Moshabela M. Implementation of a community-based intervention in the most rural and remote districts of Zambia: a process evaluation of safe motherhood action groups. *Implementation science.* 2018;13(1):74.
45. Hayes RJ, Moulton LH. *Cluster randomised trials.* Chapman and Hall/CRC; 2017.
46. Denzin NK, Lincoln YS. *The Sage handbook of qualitative research.* Sage; 2011.
47. Onyango MA, Adu-Sarkodie Y, Agyarko-Poku T, et al. "It's all about making a life": poverty, HIV, violence, and other vulnerabilities faced by young female sex workers in Kumasi, Ghana. *JAIDS Journal of Acquired Immune Deficiency Syndromes.* 2015;68:S131-S137.
48. Geibel S, King'ola N, Temmerman M, Luchters S. The impact of peer outreach on HIV knowledge and prevention behaviours of male sex workers in Mombasa, Kenya. *Sex Transm Infect.* 2012;88(5):357-362.
49. Oldenburg CE, Biello KB, Colby D, et al. Engagement with peer health educators is associated with willingness to use pre-exposure prophylaxis among male sex workers in Ho Chi Minh City, Vietnam. *AIDS patient care and STDs.* 2014;28(3):109-112.
50. Ford K, Wirawan D, Suastina W, Reed B, Muliawan P. Evaluation of a peer education programme for female sex workers in Bali, Indonesia. *International journal of STD & AIDS.* 2000;11(11):731-733.

For peer review only

For peer review only - http://bmjopen.bmj.com/site/about/guidelines.xhtml

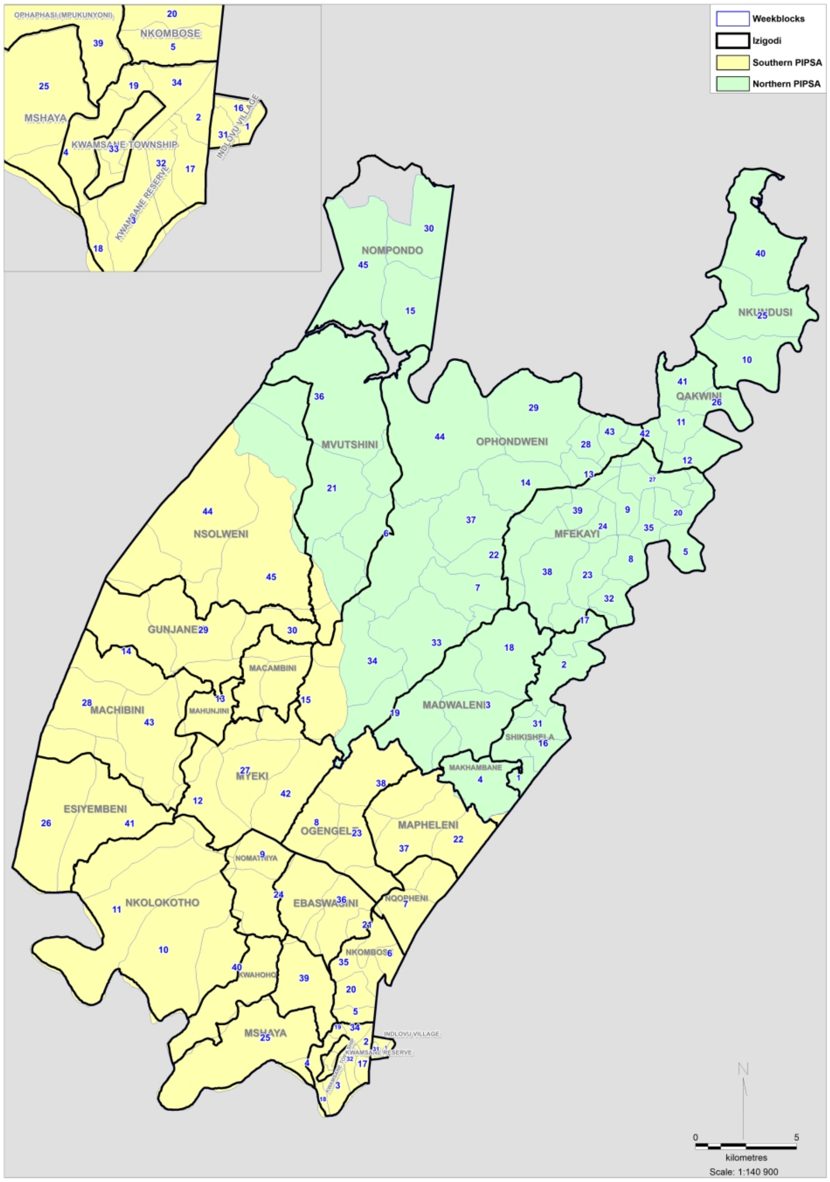

296x419mm (300 x 300 DPI)

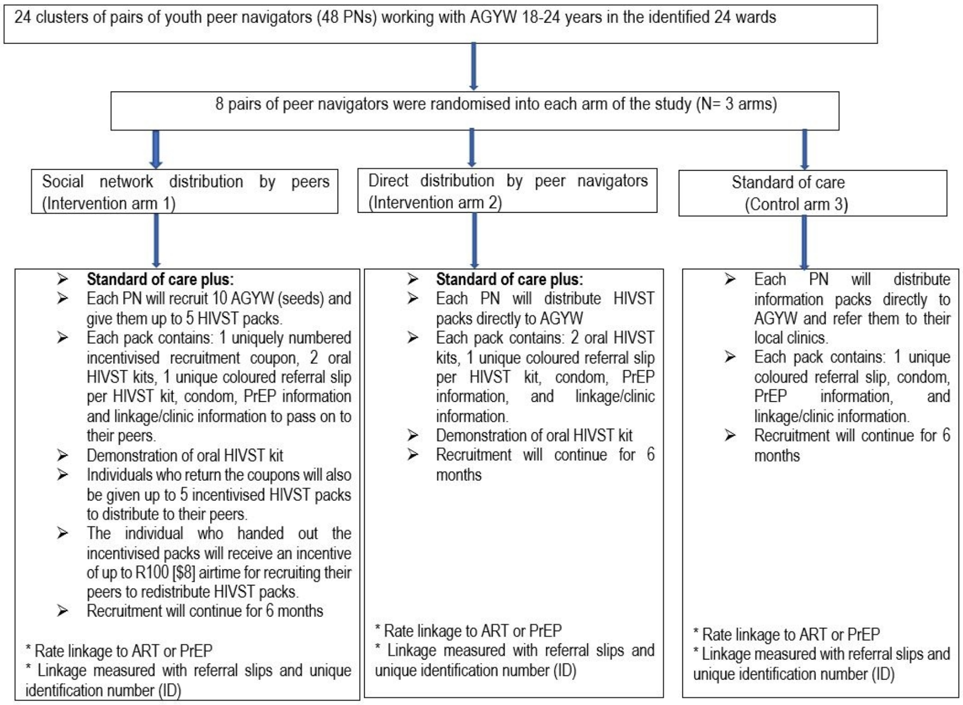

289x214mm (96 x 96 DPI)

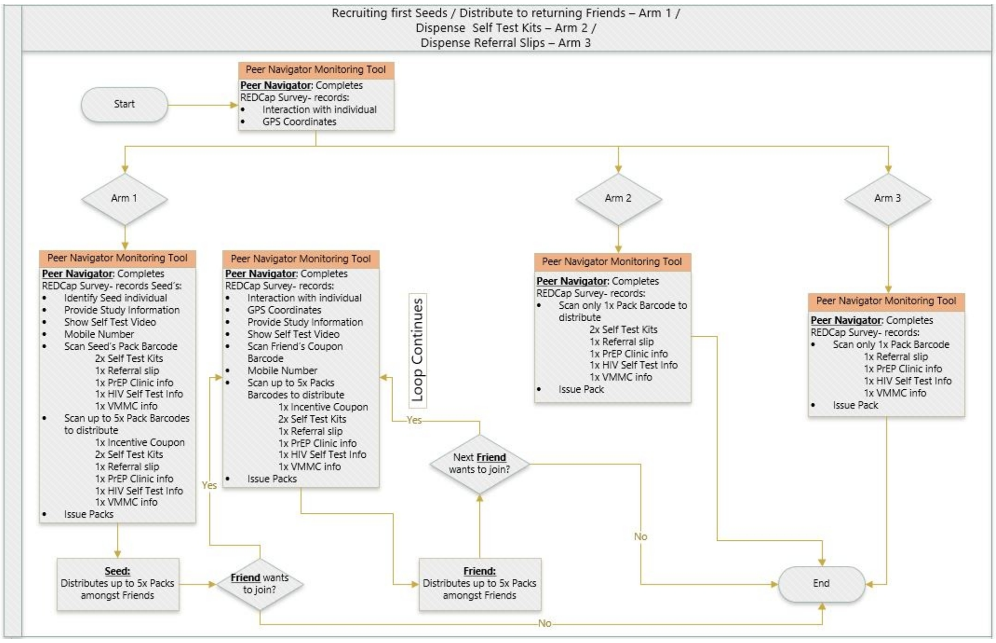

283x180mm (96 x 96 DPI)

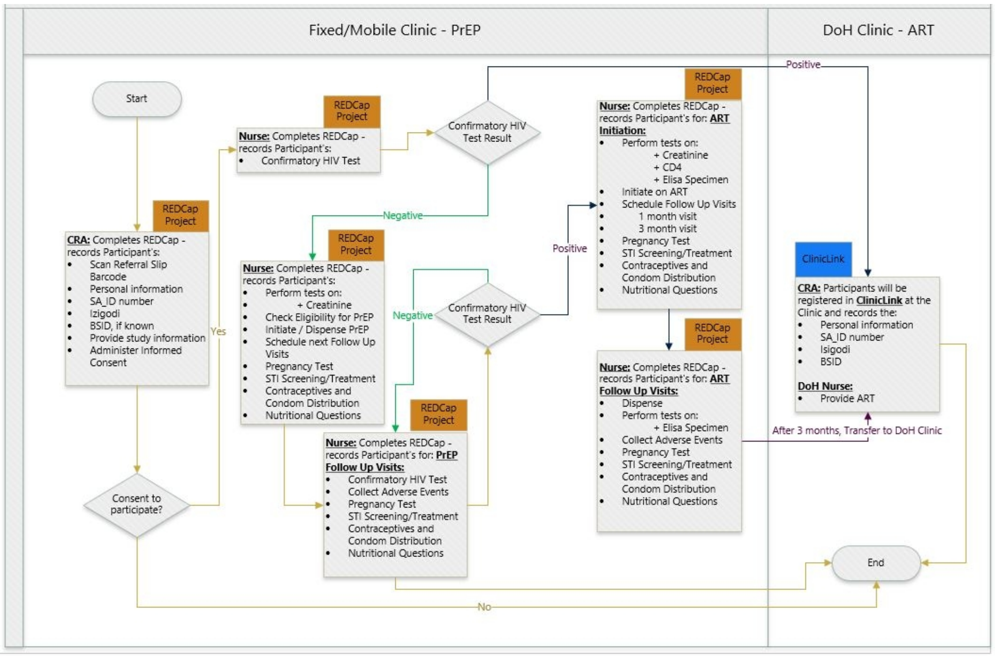

280x184mm (96 x 96 DPI)

Reporting checklist for protocol of a clinical trial.

Based on the SPIRIT guidelines.

Instructions to authors

Complete this checklist by entering the page numbers from your manuscript where readers will find each of the items listed below.

Your article may not currently address all the items on the checklist. Please modify your text to include the missing information. If you are certain that an item does not apply, please write "n/a" and provide a short explanation.

Upload your completed checklist as an extra file when you submit to a journal.

In your methods section, say that you used the SPIRIT reporting guidelines, and cite them as:

Chan A-W, Tetzlaff JM, Altman DG, Laupacis A, Gøtzsche PC, Krleža-Jerić K, Hróbjartsson A, Mann H, Dickersin K, Berlin J, Doré C, Parulekar W, Summerskill W, Groves T, Schulz K, Sox H, Rockhold FW, Rennie D, Moher D. SPIRIT 2013 Statement: Defining standard protocol items for clinical trials. Ann Intern Med. 2013;158(3):200-207

For peer review only

		Reporting Item	Page Number
**Administrative information**			
Title	#1	Descriptive title identifying the study design, population, interventions, and, if applicable, trial acronym	1

Trial registration	#2a	Trial identifier and registry name. If not yet registered, name of intended registry	2
`	#2b	All items from the World Health Organization Trial Registration Data Set	(see below for specific items)
Protocol version	#3	Date and version identifier	2
Funding	#4	Sources and types of financial, material, and other support	17
Roles and responsibilities: contributorship	#5a	Names, affiliations, and roles of protocol contributors	1, 17
Roles and responsibilities: sponsor contact information	#5b	Name and contact information for the trial sponsor	17
Roles and responsibilities: sponsor and funder	#5c	Role of study sponsor and funders, if any, in study design; collection, management, analysis, and interpretation of data; writing of the report; and the decision to submit the report for publication, including whether they will have ultimate authority over any of these activities	17
Roles and responsibilities: committees	#5d	Composition, roles, and responsibilities of the coordinating centre, steering committee, endpoint adjudication committee, data management team,	N/A

For peer review only

		and other individuals or groups overseeing the trial, if applicable (see Item 21a for data monitoring committee)	
**Introduction**			
Background and rationale	#6a	Description of research question and justification for undertaking the trial, including summary of relevant studies (published and unpublished) examining benefits and harms for each intervention	2-3
Background and rationale: choice of comparators	#6b	Explanation for choice of comparators	N/A
Objectives	#7	Specific objectives or hypotheses	4
Trial design	#8	Description of trial design including type of trial (eg, parallel group, crossover, factorial, single group), allocation ratio, and framework (eg, superiority, equivalence, non-inferiority, exploratory)	4-5
**Methods: Participants, interventions, and outcomes**			
Study setting	#9	Description of study settings (eg, community clinic, academic hospital) and list of countries where data	6

For peer review only

		will be collected. Reference to where list of study sites can be obtained	
Eligibility criteria	#10	Inclusion and exclusion criteria for participants. If applicable, eligibility criteria for study centres and individuals who will perform the interventions (eg, surgeons, psychotherapists)	8-9
Interventions: description	#11a	Interventions for each group with sufficient detail to allow replication, including how and when they will be administered	5-6
Interventions: modifications	#11b	Criteria for discontinuing or modifying allocated interventions for a given trial participant (eg, drug dose change in response to harms, participant request, or improving / worsening disease)	N/A
Interventions: adherence	#11c	Strategies to improve adherence to intervention protocols, and any procedures for monitoring adherence (eg, drug tablet return; laboratory tests)	N/A
Interventions: concomitant care	#11d	Relevant concomitant care and interventions that are permitted or prohibited during the trial	N/A
Outcomes	#12	Primary, secondary, and other outcomes, including the specific measurement variable (eg, systolic blood pressure), analysis metric (eg, change from baseline, final value, time to event), method of aggregation (eg, median, proportion), and time point for each outcome. Explanation of the clinical	10-11

For peer review only

		relevance of chosen efficacy and harm outcomes is strongly recommended	
Participant timeline	#13	Time schedule of enrolment, interventions (including any run-ins and washouts), assessments, and visits for participants. A schematic diagram is highly recommended (see Figure)	7-8
Sample size	#14	Estimated number of participants needed to achieve study objectives and how it was determined, including clinical and statistical assumptions supporting any sample size calculations	10
Recruitment	#15	Strategies for achieving adequate participant enrolment to reach target sample size	7-8
**Methods: Assignment of interventions (for controlled trials)**			
Allocation: sequence generation	#16a	Method of generating the allocation sequence (eg, computer-generated random numbers), and list of any factors for stratification. To reduce predictability of a random sequence, details of any planned restriction (eg, blocking) should be provided in a separate document that is	9

For peer review only

		unavailable to those who enrol participants or assign interventions	
Allocation concealment mechanism	#16b	Mechanism of implementing the allocation sequence (eg, central telephone; sequentially numbered, opaque, sealed envelopes), describing any steps to conceal the sequence until interventions are assigned	9
Allocation: implementation	#16c	Who will generate the allocation sequence, who will enrol participants, and who will assign participants to interventions	9
Blinding (masking)	#17a	Who will be blinded after assignment to interventions (eg, trial participants, care providers, outcome assessors, data analysts), and how	9
Blinding (masking): emergency unblinding	#17b	If blinded, circumstances under which unblinding is permissible, and procedure for revealing a participant's allocated intervention during the trial	N/A
**Methods: Data collection, management, and analysis**			
Data collection plan	#18a	Plans for assessment and collection of outcome, baseline, and other trial data, including any related processes to promote data quality (eg, duplicate measurements, training of assessors) and a	11-12

For peer review only

		description of study instruments (eg, questionnaires, laboratory tests) along with their reliability and validity, if known. Reference to where data collection forms can be found, if not in the protocol	
Data collection plan: retention	#18b	Plans to promote participant retention and complete follow-up, including list of any outcome data to be collected for participants who discontinue or deviate from intervention protocols	N/A
Data management	#19	Plans for data entry, coding, security, and storage, including any related processes to promote data quality (eg, double data entry; range checks for data values). Reference to where details of data management procedures can be found, if not in the protocol	13
Statistics: outcomes	#20a	Statistical methods for analysing primary and secondary outcomes. Reference to where other details of the statistical analysis plan can be found, if not in the protocol	13
Statistics: additional analyses	#20b	Methods for any additional analyses (eg, subgroup and adjusted analyses)	13
Statistics: analysis population and missing data	#20c	Definition of analysis population relating to protocol non-adherence (eg, as randomised analysis), and	13

For peer review only

		any statistical methods to handle missing data (eg, multiple imputation)	
**Methods: Monitoring**			
Data monitoring: formal committee	#21a	Composition of data monitoring committee (DMC); summary of its role and reporting structure; statement of whether it is independent from the sponsor and competing interests; and reference to where further details about its charter can be found, if not in the protocol. Alternatively, an explanation of why a DMC is not needed	6
Data monitoring: interim analysis	#21b	Description of any interim analyses and stopping guidelines, including who will have access to these interim results and make the final decision to terminate the trial	6
Harms	#22	Plans for collecting, assessing, reporting, and managing solicited and spontaneously reported adverse events and other unintended effects of trial interventions or trial conduct	12
Auditing	#23	Frequency and procedures for auditing trial conduct, if any, and whether the process will be independent from investigators and the sponsor	N/A
**Ethics and dissemination**			

For peer review only

Research ethics approval	#24	Plans for seeking research ethics committee / institutional review board (REC / IRB) approval	16
Protocol amendments	#25	Plans for communicating important protocol modifications (eg, changes to eligibility criteria, outcomes, analyses) to relevant parties (eg, investigators, REC / IRBs, trial participants, trial registries, journals, regulators)	N/A
Consent or assent	#26a	Who will obtain informed consent or assent from potential trial participants or authorised surrogates, and how (see Item 32)	14
Consent or assent: ancillary studies	#26b	Additional consent provisions for collection and use of participant data and biological specimens in ancillary studies, if applicable	N/A
Confidentiality	#27	How personal information about potential and enrolled participants will be collected, shared, and maintained in order to protect confidentiality before, during, and after the trial	14
Declaration of interests	#28	Financial and other competing interests for principal investigators for the overall trial and each study site	16
Data access	#29	Statement of who will have access to the final trial dataset, and disclosure of contractual agreements that limit such access for investigators	16

For peer review only

Ancillary and post trial care	#30	Provisions, if any, for ancillary and post-trial care, and for compensation to those who suffer harm from trial participation	N/A
Dissemination policy: trial results	#31a	Plans for investigators and sponsor to communicate trial results to participants, healthcare professionals, the public, and other relevant groups (eg, via publication, reporting in results databases, or other data sharing arrangements), including any publication restrictions	14
Dissemination policy: authorship	#31b	Authorship eligibility guidelines and any intended use of professional writers	N/A
Dissemination policy: reproducible research	#31c	Plans, if any, for granting public access to the full protocol, participant-level dataset, and statistical code	N/A
**Appendices**			
Informed consent materials	#32	Model consent form and other related documentation given to participants and authorised surrogates	See Supplementary file
Biological specimens	#33	Plans for collection, laboratory evaluation, and storage of biological specimens for genetic or molecular analysis in the current trial and for future use in ancillary studies, if applicable	N/A

For peer review only

None The SPIRIT checklist is distributed under the terms of the Creative Commons Attribution License CC-BY-ND 3.0. This checklist can be completed online using https://www.goodreports.org/, a tool made by the EQUATOR Network in collaboration with Penelope.ai

For peer review only
